# GT-00AxIL15, a Novel Tumor-Targeted IL-15-Based Immunocytokine for the Treatment of TA-MUC1-Positive Solid Tumors: Preclinical In Vitro and In Vivo Pharmacodynamics and Biodistribution Studies

**DOI:** 10.3390/ijms25031406

**Published:** 2024-01-24

**Authors:** Johanna Gellert, Anika Jäkel, Antje Danielczyk, Christoph Goletz, Timo Lischke, Anke Flechner, Laura Dix, Alexandra Günzl, Patrik Kehler

**Affiliations:** 1 Glycotope GmbH, Robert-Roessle-Str.10, 13125 Berlin, Germanyantje.danielczyk@glycotope.com (A.D.); timo.lischke@glycotope.com (T.L.); patrik.kehler@glycotope.com (P.K.); 2 Karyon Biopharma Consulting e.U., Brunn am Gebirge, Austria

**Keywords:** interleukin-15, TA-MUC1, monoclonal antibody, immunocytokine, cancer, immunotherapy, anti-tumor cytotoxicity

## Abstract

GT-00AxIL15 is a novel interleukin-15-based immunocytokine targeting a tumor-specific, glycosylated epitope of MUC1 (TA-MUC1). We characterized mode of action, pharmacokinetic (PK) and pharmacodynamic (PD) properties and investigated the relevance of TA-MUC1 binding for the concept of delivering IL-15 to solid tumors. In vitro pharmacology was analyzed in binding and cell-based assays. The in vivo PK profile and IL-15-mediated PD effects of GT-00AxIL15 were investigated in tumor-free mice. Tumor accumulation, immune infiltration and anti-tumor activity were assessed in TA-MUC1+ syngeneic and xenogeneic murine tumor models. GT-00AxIL15 was shown to specifically bind TA-MUC1 on tumor cells via its mAb moiety, to IL-15 receptors on immune cells via its IL-15 fusion modules and to FcγRs via its functional Fc-part. In vitro, NK, NKT and CD8+ T cells were activated and proliferated, leading to anti-tumor cytotoxicity and synergism with antibody-dependent cellular cytotoxicity (ADCC)-mediating mAbs. In vivo, GT-00AxIL15 exhibited favorable PK characteristics with a serum half-life of 13 days and specifically accumulated in TA-MUC1+ tumors. In the tumor microenvironment, GT-00AxIL15 induced robust immune activation and expansion and mediated anti-metastatic and anti-tumor effects in syngeneic and xenograft tumor models. These results support the rationale to improve PK and anti-tumor efficacy of IL-15 by increasing local concentrations at the tumor site via conjugation to a TA-MUC1 binding mAb. The tumor-selective expression pattern of TA-MUC1, powerful immune activation and anti-tumor cytotoxicity, long serum half-life and tumor targeting properties, render GT-00AxIL15 a promising candidate for treatment of solid tumors with high medical need, e.g., ovarian, lung and breast cancer.

## 1. Introduction

Interleukin-15 (IL-15) is a potent pro-inflammatory cytokine of the common gamma-chain (γc) family playing a crucial role in the differentiation and proliferation of NK and T cells and in the survival of CD8+ memory T cells [1]. It signals through the heterotrimeric interleukin-15 receptor (IL-15R) consisting of the IL-15Rα and IL-15Rβγ subunits, whereas the IL-15/IL-15Rα complex is presented to IL-15Rβγ on the same (cis) or adjacent (trans) cells [2,3]. IL-15 shares the βγ receptor and pro-inflammatory potential on T and NK cells with IL-2. However, it neither activates regulatory T cells, inhibits immune responses through activation-induced cell death nor induces vascular capillary leak syndrome like IL-2 [1]. Therefore, it was ranked as top immunotherapeutic agent by the National Cancer Institute in 2008 [4]. However, clinical trials demonstrated that recombinant human IL-15 (rhIL-15) is limited by its short half-life of only a few hours and the observed dose-limiting toxicities, which prevent clinical benefit as monotherapy [5,6,7,8].

To address these problems, several IL-15-based agonists have been developed to improve pharmacokinetics and efficacy while limiting toxicities [9,10]. Several half-life extension technologies exist to increase size and reduce renal clearance of IL-15 including dimerization with parts of IL-15Rα, attachment of polyethylene glycol (PEG) or generation of immunocytokines by fusing interleukins to an antibody backbone [11]. The latter enables neonatal fragment crystallizable receptor (FcRn) recycling to reduce serum clearance thereby achieving sustained IL-15 exposure for improved effector cell activation.

Most of these first-generation constructs, e.g., SOT101/nanrilkefusp alfa [12,13], N-803/nogapendekin alfa [14,15,16] and NIZ985 [5,17] apply the concept of IL-15 superagonism by complexing IL-15 with a part of IL-15Rα, not only for half-life extension, but also for increased efficacy, because preclinical studies indicate that the IL-15/IL-15Rα dimer, rather than the IL-15 monomer, is more bioactive when trans-presented to NK and CD8+ memory T cells [18,19,20]. However, recent developments move away from these highly potent superagonists to avoid attenuated biological responsiveness of immune effector cells as observed for N-803 after multiple dosing [15]. XmAb24306 and NKTR-255 have reduced IL-15R binding affinities to achieve moderate potency, sustained lymphocyte stimulation and improved PK [21,22]. In general, these first-generation immunocytokines show promising results in the clinic, but lack a tumor-targeting approach and are still challenged by their limited bioavailability in vivo [11].

Antibody fusions can not only be used for half-life extension, but also as “vehicles” for the delivery of cytokines to the disease site by Fragment antigen binding (Fab) mediated recognition to a defined antigen, but so far only few targeted approaches for IL-15 are in early stage clinical (KD-033, IAP0971) [23,24], or preclinical development (N-809, N-820, SOT201 or LAG3xIL15) [25,26,27,28]. However, their mAb targets are not tumor-specific considering the broad expression of PD-L1, PD-1, CD20 or LAG3 on healthy cells.

We developed GT-00AxIL15 as first-in-class IL-15-based immunocytokine targeting a tumor-associated, glycosylated epitope of MUC1 (TA-MUC1) to optimize tumor accumulation, efficacy, safety and half-life of IL-15 compared to untargeted therapeutics. The antibody-targeting moiety of GT-00AxIL15 is based on GT-00A (gatipotuzumab, historically PankoMab), a humanized IgG1 mAb designed for exclusive binding to TA-MUC1 and clinically developed for treatment of solid tumors [29,30,31]. TA-MUC1 is a combined carbohydrate-protein epitope which is expressed on a variety of carcinomas, their respective metastases as well as on cancer stem cells and is virtually absent on normal cells [32,33,34,35,36,37,38,39]. In order to promote both innate and adaptive immune response against tumors, GT-00AxIL15 contains two human wild-type IL-15 molecules linked to the Fc-domain of the mAb. Importantly, the IL-15 moiety is not modified or precomplexed to IL-15Rα to retain binding properties of natural IL-15.

Here we report preclinical in vitro and in vivo studies with GT-00AxIL15 to characterize its MoA and explore its PK and PD effects. Our results support the potential of GT-00AxIL15 as next-generation tumor-targeted IL-15-based immunocytokine for treatment of solid tumors.

## 2. Results

### 2.1. GT-00AxIL15 Specifically Binds to Its Targets TA-MUC1, IL-15R and FcγRIIIa on Human Tumor Cell Lines and Primary Immune Cells

**Figure 1:**
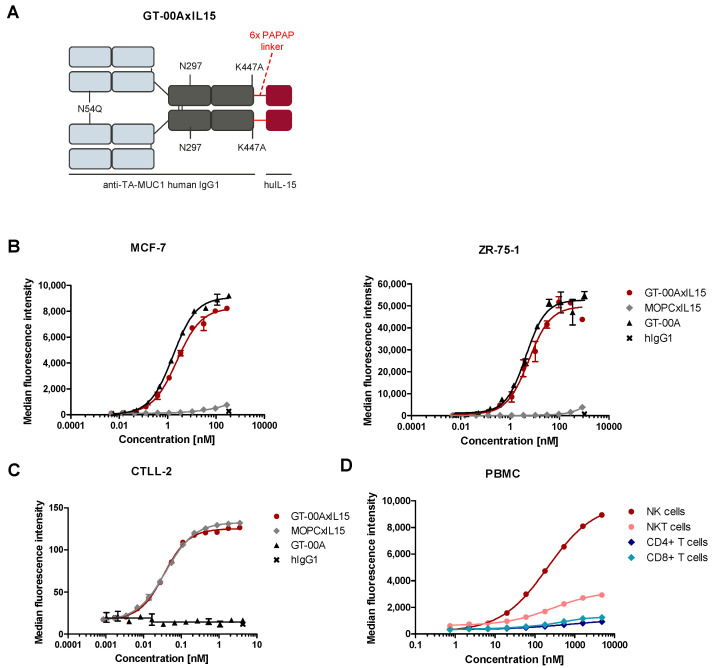
GT-00AxIL15 binds to its targets TA-MUC1 and IL-15R on human tumor cell lines and immune cells. (**A**) Structure of GT-00AxIL15: Fc- (dark grey) and Fab- (light grey) part of the parental mAb GT-00A, human IL-15 (dark red), N54Q mutation to remove N-glycosylation site in the Fab domain, Fc N-glycosylation site N297, K447A mutation at transition sequence to IL-15 modules to remove potential protease site; red line depicts 6xPAPAP linker between IL-15 and the heavy chain. (**B**) Binding to cellular TA-MUC1 on human MCF-7 and ZR-75-1 tumor cell lines. (**C**) Binding to cellular IL-15R on murine CTLL-2 cell line. (**D**) Binding of AF647-labeled GT-00AxIL15 to human NK, NKT, CD4+ and CD8+ T cells of a representative PBMC donor. Mean ± SD (*n* = 2) of representative experiments.

The structure of GT-00AxIL15 and its three functional domains to bind TA-MUC1, IL-15R and FcγR are depicted in Figure 1A. Several experiments were performed to investigate the binding properties of GT-00AxIL15 to these targets. In ELISA studies using different MUC1 peptides, GT-00AxIL15 and its parental mAb GT-00A, but not the untargeted MOPCxIL15 isotype control, bound to the glycosylated MUC1 peptide (Figure S1A). GT-00AxIL15 does not bind to the non-glycosylated peptide confirming glycan-dependency as hallmark of tumor-selective MUC1 binding (Figure S1B). The affinity of GT-00AxIL15 for TA-MUC1 was determined by fluorescence proximity sensing as 3.18 nM (Table S3). Further, binding to IL-15Rɑ (Figure S1C) and IL-15Rβ (Figure S1D) was observed for GT-00AxIL15 and MOPCxIL15, but not for GT-00A lacking the IL-15 fusion. As expected, in a dual binding ELISA, only GT-00AxIL15, but not the controls showed simultaneous binding to TA-MUC1 and IL-15Rɑ (Figure S1E). Finally, it was shown that GT-00AxIL15 as well as MOPCxIL15 and GT-00A bound to FcγRIIIa (Figure S1F), indicating that Fc functionality is not impacted by fusion of the IL-15 modules to the Fc-part of the mAb moiety.

The results were confirmed in cellular binding assays (Figure 1). GT-00AxIL15 and GT-00A, but not MOPCxIL15 or hIgG1 isotype control bound to TA-MUC1 on human tumor cell lines MCF-7 and ZR-75-1 (Figure 1B). Only GT-00AxIL15 and MOPCxIL15 bound to IL-15R on CTLL-2 cells (Figure 1C). Within primary PBMC preparations, it was shown that GT-00AxIL15 bound strongest to human NK cells followed by NKT, CD8+ and CD4+ T cells (Figure 1D).

In conclusion, these data confirm specific binding of GT-00AxIL15 via its three functional domains and suggest that simultaneous binding to TA-MUC1 on tumor cells and IL-15R on immune cells is possible.

### 2.2. GT-00AxIL15 Induces Proliferation of a Human NK Cell Line and Potently Activates and Expands Primary Immune Effector Cells In Vitro

**Figure 2:**
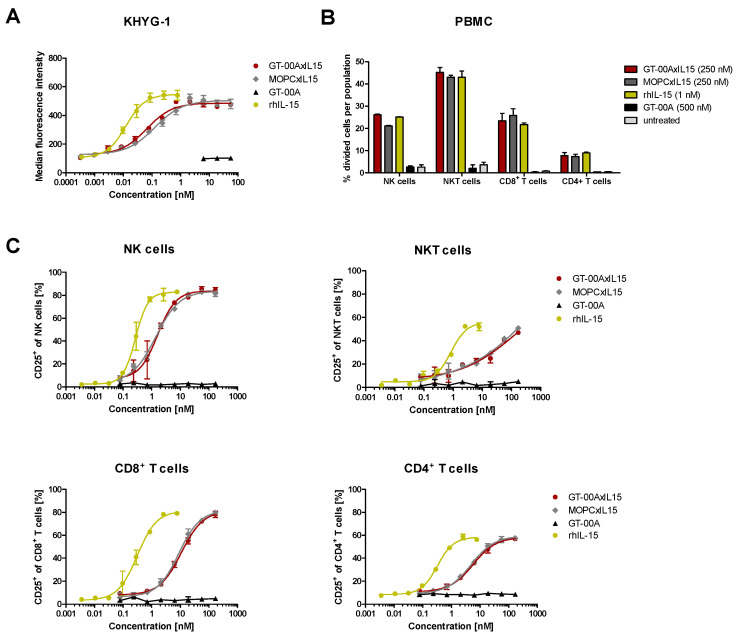
GT-00AxIL15 induces proliferation of human NK cell line and primary PBMCs and activates human immune effector cells. (**A**) IL-15 bioactivity assay: Proliferation of KHYG-1 cells after 48 h treatment with test antibodies as determined by viability assay; mean ± SD (*n* = 3) from a representative experiment. (**B**) Proliferation and (**C**) activation of primary human immune cell subsets determined by flow cytometry. Mean ± SD (*n* = 2) from representative experiments.

In an IL-15 bioactivity assay, GT-00AxIL15 induced proliferation of the IL-15R-expressing human NK cell line KHYG-1 comparable to MOPCxIL15 whereas GT-00A had no effect (Figure 2A). GT-00AxIL15 also induced downstream-signaling by activation of signal transducer and activator of transcription 5 (STAT5) (Figure S2) and proliferation of primary human immune cells, i.e., NK, NKT and both CD8+ and CD4+ T cells (Figure 2B). As shown by upregulation of CD25, GT-00AxIL15 effectively activated these cells with NK cells reacting most sensitively (Figure 2C). In signaling, proliferation and activation assays, the potency of GT-00AxIL15 was lower compared to same molar concentrations of rhIL-15, but efficacy, i.e., the maximum achievable effect, was comparable. Overall, experiments confirmed the successful induction of immune cell proliferation through the IL-15 moiety of GT-00AxIL15, as GT-00A lacking signaling via IL-15 was not able to induce any proliferation. Furthermore, it was shown by immunophenotyping of human PBMCs that GT-00AxIL15 induces expression of several immune checkpoint molecules on T cell subsets (e.g., OX-40, ICOS, Tim-3, TIGIT, PD-1) and NK(T) cells (NKp46, NKG2A) (Table S4) indicating full activation and providing rationale for combination approaches.

### 2.3. GT-00AxIL15 Mediates Tumor Immune Infiltration and Elicits Enhanced Anti-Tumor Cytotoxicity In Vitro

A 3D co-culture assay of PBMCs with TA-MUC1+ MCF-7 tumor spheroids was performed to investigate effects of GT-00AxIL15 on tumor immune infiltration. Numbers of CD3+ (Figure S3A) and CD8+ (Figure S3B) lymphocytes in tumor spheroids increased after treatment with GT-00AxIL15, but not the controls. NK cells could not be investigated for technical reasons.

**Figure 3:**
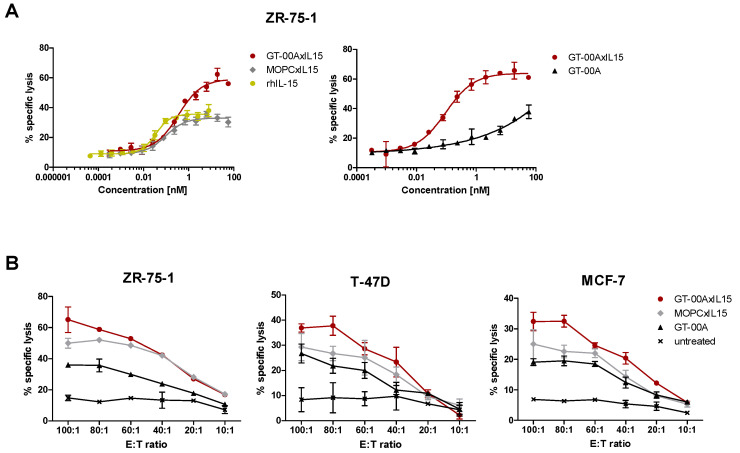
GT-00AxIL15 shows enhanced anti-tumor cytotoxicity compared to untargeted IL-15 variants and parental anti-TA-MUC1 mAb. Europium release assays using TA-MUC1+ cell lines as target cells in the presence of primary human PBMCs as effector cells to determine anti-tumor cytotoxicity (mean ± SD; *n* = 3 from representative experiments) with test items titrated at fixed E:T ratio (80:1) in ZR-75-1 cells (**A**) or titration of E:T ratio at fixed test item concentration (20 nM) in ZR-75-1 (left), MCF-7 (mid) and T-47D (right) tumor cells (**B**).

In europium release assays with human PBMC as effector cells, GT-00AxIL15 induced lysis of ZR-75-1 cells (fixed E:T ratio of 80:1) at much lower concentrations than GT-00A-mediated ADCC and with a higher maximum compared to MOPCxIL15 and rhIL-15 (Figure 3A). In a further set of experiments with fixed test item concentration and varying E:T ratios, tumor cell lysis was induced in all three tested cell lines (ZR-75-1, MCF-7 and T-47D) by GT-00AxIL15 and controls, but again, GT-00AxIL15-mediated effects were higher compared to the ADCC induced by GT-00A or the IL-15-induced cytotoxicity of MOPCxIL15 (Figure 3B). This confirms that the enhanced cytotoxic effects observed for GT-00AxIL15 are mediated by an ADCC effect via TA-MUC1-targeting in combination with IL-15-induced immune cell-mediated cytotoxicity of T and NK cells. Further, we could show synergistic effects on tumor cell lysis by combination of GT-00AxIL15 with approved ADCC-mediating mAbs cetuximab (αEGFR) and avelumab (αPD-L1) (Figure S4A,B).

### 2.4. GT-00AxIL15 Elicits a Prolonged Half-Life and Induces Expansion of NK, NKT and CD8+ T Cells In Vivo after Single and Multiple Injections in Tumor-Free Mice

**Figure 4:**
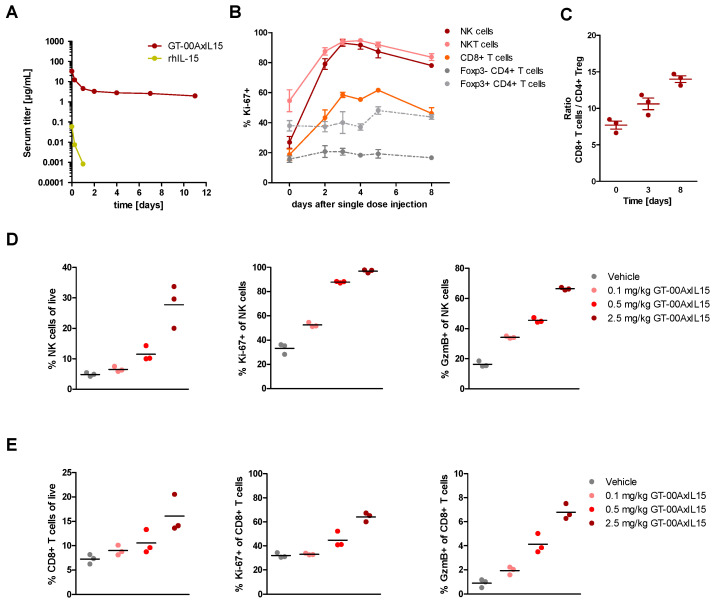
GT-00AxIL15 has a prolonged half-life and induces activation and expansion of NK, NKT and CD8+ effector T cells in tumor-free mice. (**A**) Mouse PK profiles after single i.v. administration of 2 mg/kg GT-00AxIL15 or equivalent dose of rhIL-15 (0.29 mg/kg). (**B**,**C**) PD effects in spleen after single i.v. injection of 1 mg/kg GT-00AxIL15: Relative proportions of (**B**) Ki-67+ cells in NK, NKT, CD8+, Foxp3-CD4+ and Foxp3+CD4+ T cells after d3 and (**C**) ratio of CD8+ T effector cells to CD4+Foxp3+ regulatory T cells. (**D**,**E**) Dose-dependency of PD effects on peripheral blood NK (**D**) and CD8+ T cells (**E**) as determined by relative expansion (left), Ki-67 (middle) and GzmB (right) expression after single doses of up to 2.5 mg/kg GT-00AxIL15. Mean ± SEM (*n* = 3 mice).

The short half-life of rhIL-15 and IL-15 agonists ranging from few hours to 1–2 days [11] is one of the factors limiting their clinical potential. In contrast, for GT-00AxIL15, a superior terminal serum half-life of 13 ± 3.1 days was determined in mice after single i.v. administration, whereas rhIL-15 was very rapidly cleared from serum as expected (Figure 4A).

We then investigated PD effects of the IL-15 modules of GT-00AxIL15 on immune effector cells in tumor-free mice. Upon single injection of 1 mg/kg GT-00AxIL15, expression of Ki-67 was strongly up-regulated, peaking around day 3 and not reaching baseline levels until day 8, in all tissues evaluated (spleen, blood and ingLN) for NK, NKT and CD8+ T cells, while CD4+ T cells barely increased (Figure 4B and Figure S5A). This selective expansion of effector T cells led to an ultimate increase of the effector to regulatory T cell ratio (CD8+/CD4+Foxp3+) in spleen (Figure 4C).

In a second PD study in tumor-free mice, a dose-dependent increase of proliferation marker Ki-67 expression in NK and CD8+ T cells translated into a relative expansion of both subsets in blood after single dose treatment of 0.1–2.5 mg/kg GT-00AxIL15 (Figure 4D,E). Especially in NK cells but also in CD8+ T cells, Granzyme B (GzmB) expression increased in a dose-dependent manner, which is of interest as the release of this protease is one of the major mechanisms of cytotoxic cells to induce apoptosis in target cells.

Given that for IL-15 superagonists, lymphocyte exhaustion and reduced responsiveness after multiple treatments were observed [15], effects of a second GT-00AxIL15 dose on NK (Figure S5B) and CD8+ T cells (Figure S5C) were investigated compared to single dosing. With comparable kinetics, Ki-67 expression declined to baseline levels at d14 after both a single and second dose of 2.5 mg/kg GT-00AxIL15 (d0/7). Similarly, repeated stimulation re-expanded the NK cell population to the same extent as single dose treatment and even further increased the CD8+ T cell population. Also, GzmB expression of NK and CD8+ T cells could be re-induced with a second dose of GT-00AxIL15 to the same extent as after first administration.

### 2.5. GT-00AxIL15 Accumulates in TA-MUC1+ Tumors, Selectively Expands Tumor-Infiltrating Lymphocytes (TIL) and Reduces Primary Tumors and Metastatic Load In Vivo

**Figure 5:**
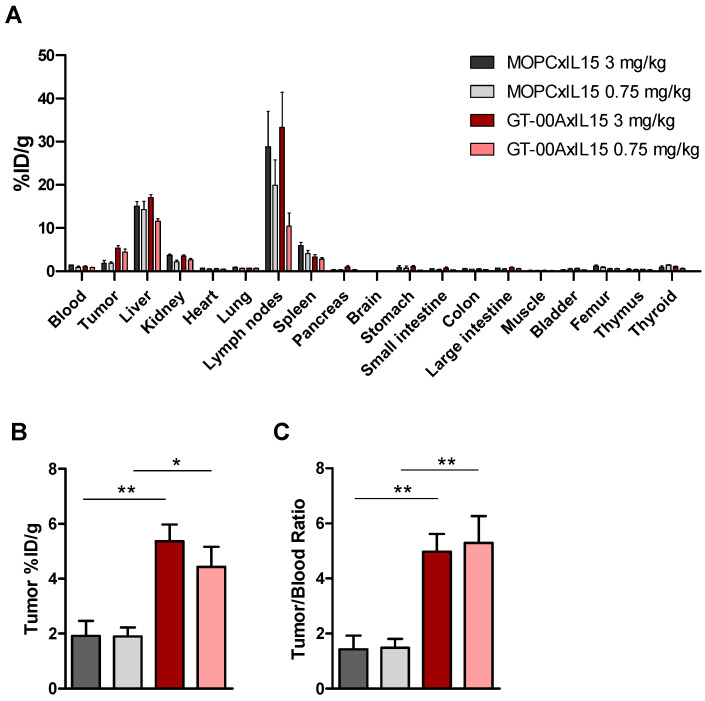
GT00AxIL15 shows improved tumor accumulation compared to an untargeted immunocytokine. (**A**) Biodistribution in respective organs and tissues (in %ID/g) at final timepoint (72 h) after i.v. injection of ^89^Zr-GT-00AxIL15 and ^89^Zr-MOPCxIL15 into hMUC1-B16.F10 tumor-bearing mice. (**B**) Tumor accumulation and (**C**) Tumor-to-Blood ratio at 72 h; One-Way ANOVA, * *p* < 0.05, ** *p* < 0.01, Mean ± SEM from *n* = 5 mice.

To investigate tumor accumulation and relevance of TA-MUC1 binding for biodistribution, GT-00AxIL15 and its untargeted control MOPCxIL15 were subjected to a PET imaging study. 72 h after i.v. injection into hMUC1-B16.F10 tumor-bearing mice, ^89^Zr-labelled GT-00AxIL15 was detected in the TA-MUC1+ tumor as well as in lymphoid organs (spleen and lymph nodes) and in the liver indicating clearance via the hepatobiliary pathway as described for other IL-15 agonists [15] (Figure 5A). Importantly, uptake of ^89^Zr-GT-00AxIL15 in the tumor was significantly higher compared to the untargeted ^89^Zr-MOPCxIL15 construct at both doses (high dose 5.4% vs. 1.9%, low dose 4.4% vs. 1.9%) (Figure 5B), confirming that TA-MUC1 binding improves tumor accumulation. Clearance from blood and specific accumulation in the tumor is reflected by significantly higher tumor-to-blood ratios for ^89^Zr-GT-00AxL15 compared to ^89^Zr-MOPCxIL15 (high dose 5.0 vs. 1.4, low dose 5.3 vs. 1.5) (Figure 5C).

**Figure 6:**
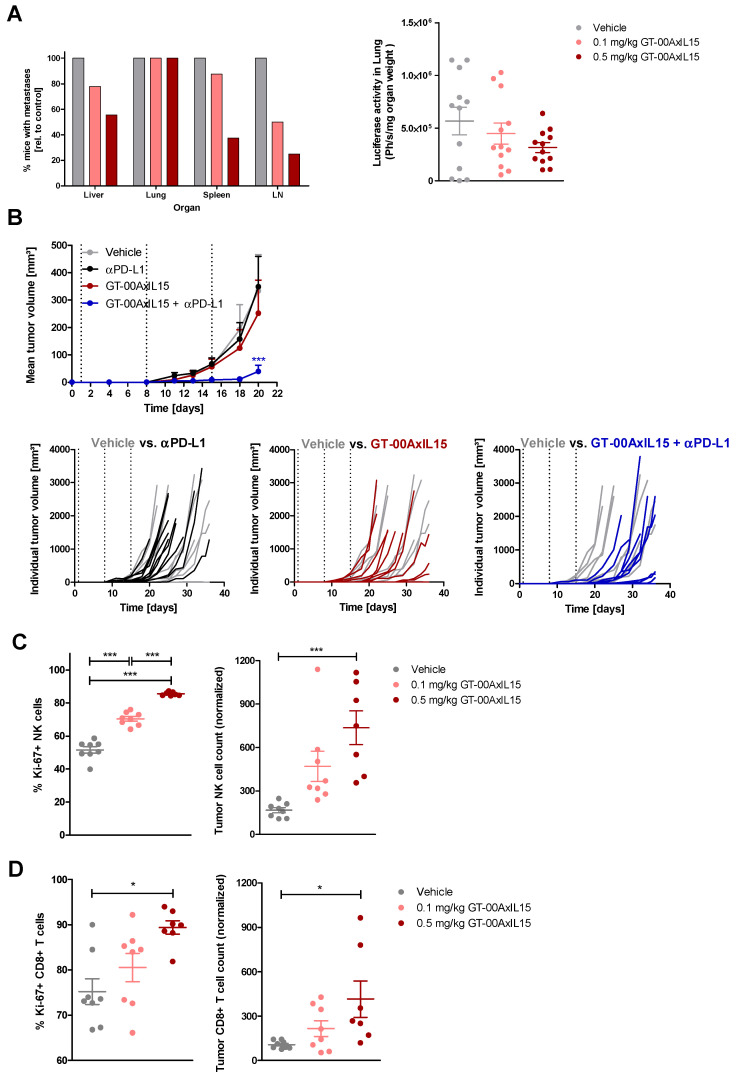
GT-00AxIL15 shows in vivo efficacy in syngeneic hMUC1-B16.F10 tumor models. (**A**) Metastasis model (i.v.): mice were treated with vehicle, 0.1 mg/kg or 0.5 mg/kg GT-00AxIL15 i.v. on d1 and 8. On d15, organs were collected and analyzed; percentage of organs containing metastases relative to vehicle group (left) and luciferase activity in lung (mean ± SEM, *n* = 12) (right). (**B**) Solid tumor model (mfp): TV until day 20 (mean ± SEM, *n* = 12), and corresponding spider plots after treatment with vehicle, 0.5 mg/kg GT-00AxIL15, 200 µg/mouse anti-PD-L1 antibody, or combination thereof on d1, 8 and 15 (dotted vertical lines); 2-way ANOVA, Bonferroni post-test, *** *p* < 0.001. (**C**,**D**) Proliferation and expansion of tumor-infiltrating NK and CD8+ T cells in solid tumor model (mfp): Mice with established tumors (~130 mm^3^) were treated once with vehicle, 0.1 mg/kg or 0.5 mg/kg GT-00AxIL15 i.v.; Percentage of Ki-67+ cells and absolute counts of NK cells (**C**) and CD8+ T cells (**D**) in tumors after d3; Mean ± SEM from *n* = 7–8 mice, 1-way ANOVA * *p* < 0.05; *** *p* < 0.001.

We next investigated anti-tumor effects of GT-00AxIL15 in different syngeneic TA-MUC1+ tumor models. In a hMUC1-B16.F10 melanoma metastasis model, GT-00AxIL15 treatment with 0.1 and 0.5 mg/kg dose-dependently reduced the number of mice with metastases in the liver, spleen and lymph nodes. Lung metastases were identified in all mice, however, the metastatic load in lung, evaluated via bioluminescence imaging, showed a trend towards a dose-dependent reduction upon treatment, although not reaching statistical significance. This suggests that GT-00AxIL15 can reduce spontaneous metastasis formation in vivo (Figure 6A).

In a hMUC1-B16.F10 solid tumor model, neither 0.5 mg/kg GT-00AxIL15 nor ɑPD-L1 alone resulted in significant inhibition of primary tumor growth compared to the vehicle control. However, the combination of GT-00AxIL15 with ɑPD-L1 resulted in a significant growth inhibition of this very aggressively growing tumor (Figure 6B).

To characterize the effects on TILs in this model, mice with established hMUC1-B16.F10 tumors were treated with a single dose of 0.5 mg/kg GT-00AxIL15. After three days, treatment effects on absolute TV were not yet visible (data not shown) but flow cytometric analysis of tumors revealed a statistically significant and dose-dependent increase of %Ki-67-positive NK and CD8+ T cells translating into higher absolute cell counts within the tumor (Figure 6C,D). This confirms that PD effects of GT-00AxIL15 on immune cells observed in vitro are also exerted in vivo. Remarkably, expansion of NK and CD8+ T cells was more prominent in the tumor than in the periphery. NK cell numbers expanded 4.6-fold within the tumor and 1.6-fold in the spleen, whereas CD8+ T cells showed an expansion of 3.7-fold within the tumor and only 1.4-fold in the spleen (Table S5). Finally, also in the hMUC1-CT26.wt colorectal cancer model, 0.1 mg/kg GT-00AxIL15 significantly delayed tumor growth (Figure S6A,C). The treatment effect of 0.1 mg/kg GT-00AxIL15 was in the same range as anti-PD-L1 monotherapy and combination with anti-PD-L1 had no impact on TV, but further prolonged survival (Figure S6B).

In conclusion, we could confirm anti-tumor effects of GT-00AxIL15 in monotherapy and in combination with anti-PD-L1 in different syngeneic TA-MUC1+ tumor models.

### 2.6. GT-00AxIL15 Shows In Vivo Efficacy and Elicits PD Effects in a Humanized DU-145 Prostate Cancer Model

**Figure 7:**
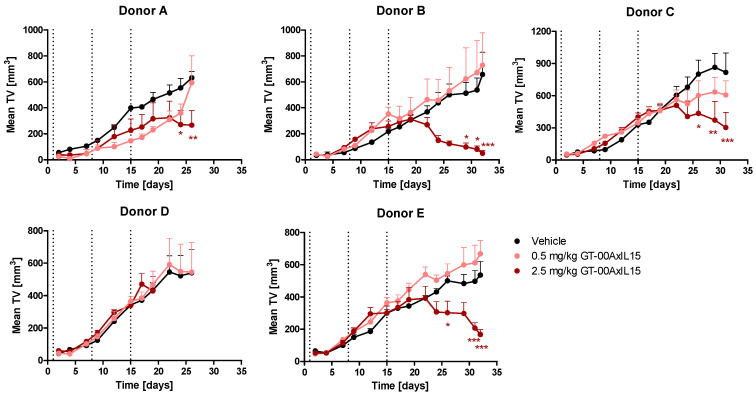
GT-00AxIL15 shows in vivo efficacy in humanized DU-145 prostate cancer model (challenge setting). DU-145 tumor cells and PBMC from 5 donors were mixed and implanted in NCG mice. Tumor volume (mean ± SEM, *n* = 3–4) after i.v. treatment with GT-00AxIL15 or vehicle on d1, 8 and 15 (dotted vertical lines); 2-way ANOVA, Bonferroni post-test, * *p* < 0.05, ** *p* < 0.01, *** *p* < 0.001.

We next investigated anti-tumor effects in PBMC-humanized DU-145 tumor-bearing mice. For 4/5 PBMC donors, repeated injections of up to 2.5 mg/kg GT-00AxIL15 induced a significant reduction of TV (Figure 7), with disease stabilization followed by regression starting on day 19 (4 days after the third administration) in all responsive donors. Highest anti-tumor activity expressed by tumor growth inhibition (TGI) (Table 1) was found for the high dose of 2.5 mg/kg and ranged from 57.7% (Donor A) up to 92.5% (Donor B). Treatment with 0.5 mg/kg induced moderate TGI of 25.6% only in donor C.

In the therapeutic setting against established tumors, TV was not reduced, but even increased after treatment with 2.5 mg/kg GT-00AxIL15 in two donors (Figure S7A; trend in two additional donors). Tumor analysis on a single cell level at study end revealed a profound infiltration of human CD45+ immune cells, mainly hCD56- CD3+ T cells but also hCD56+ NK(T) cells (Figure S7B), leading to a reduction of hCD45- mCD45- tumor cells from 67.5% to 36% (donor B) and from 75% to 57% (donor C) compared to control (Figure S7C).

## 3. Discussion

GT-00AxIL15 is a novel immunocytokine designed to direct the highly potent immune cell stimulator IL-15 to the tumor and its microenvironment via its TA-MUC1-targeting mAb moiety thereby improving its PK/PD properties and anti-tumor efficacy. The approach of fusing IL-15 to mAbs or their fragments has been increasingly pursued in the last years, however, the targets of these next-generation immunocytokines (e.g., PD-1, PD-L1, LAG3, CD20) are not tumor-specific, but rather broadly expressed on healthy cells. In contrast, GT-00AxIL15 targets a combined carbohydrate-protein epitope on MUC1 which is specifically expressed on a variety of carcinomas, their respective metastases as well as on cancer stem cells and is virtually absent on normal cells [32,33,34,35,36,37,38,39].

Our data support that GT-00AxIL15 specifically binds the glycosylated but not the non-glycosylated MUC1 peptide with high affinity, confirming glycan-dependency as hallmark of tumor-selective MUC1 binding [32,36,37]. GT-00AxIL15 simultaneously binds TA-MUC1 as tumor target and IL-15R for immune activation, retaining wild-type IL-15 binding characteristics, i.e., binding to endogenous IL-15Rα and IL-15Rβγ, resulting in natural downstream-signaling and IL-15-mediated effects such as induction of activation and proliferation of immune effector cells. Its moderate IL-15R affinity and lower potency compared to IL-15 superagonists and even rhIL-15 is considered advantageous, as high affinity IL-15R binding would have negatively interfered with TA-MUC1-mediated tumor accumulation due to fast clearance by a cytokine sink [11]. The construct design was based on the rationale, that by simultaneous binding to TA-MUC1, higher IL-15 doses could be applied to accumulate in the tumor, without leading to lymphocyte exhaustion and reduced responsiveness after multiple treatments as described for IL-15 superagonists [15].

As GT-00AxIL15 with its three functional domains binds TA-MUC1, IL-15R and FcyRIIIa, rather complex in vitro assays were required to elucidate its multifold MoA and carve out all its PD effects in comparison to untargeted IL-15 in vitro. We could show that GT-00AxIL15 induced TA-MUC1-driven immune cell infiltration into 3D tumor spheroids. Further, GT-00AxIL15 exerted enhanced anti-tumor cytotoxicity compared to its parental mAb GT-00A and an untargeted MOPCxIL15 isotype control, which is mediated by TA-MUC1-targeting in combination with increased effector cell-mediated cytotoxicity induced by the IL-15 modules. GT-00AxIL15 had a favorable PK profile in mice with a considerably longer half-life compared to rhIL-15 and other IL-15R agonists (13 days vs. few hours up to <2 days) [11]. Such a profound half-life extension was so far only achieved by Xencor’s approach to engineer for attenuated IL-15R affinity (XmAb24306) [21] and may improve patient convenience due to less frequent dosing.

In PD studies in tumor-free mice, GT-00AxIL15 induced substantial and sustained proliferation and activation of NK, NKT and CD8+ T cells, responses were dose-dependent and could be maintained by weekly dosing. Stable expression of GzmB in cytotoxic immune effector cells further supports our rationale to use a potency-reduced IL-15 fusion to circumvent exhaustion. Further, we used different syngeneic mouse models inoculated with human TA-MUC1-transfected tumor cell lines to study biodistribution and anti-tumor effects of GT-00AxIL15 in vivo. GT-00AxIL15 was cleared via the hepatobiliary pathway as described for other IL-15 agonists [15] and importantly, as shown by direct comparison to an untargeted isotype control, was not only found in immune cell-containing lymphoid organs, but specifically accumulated in TA-MUC1+ tumors. In the hMUC1-B16.F10 melanoma metastasis model, a reduction of metastatic load was observed. Against solid hMUC1-B16.F10 tumors, GT-00AxIL15 monotherapy did not inhibit primary tumor growth, but the expected PD effects on TILs, i.e., significant expansion of NK and CD8+ T cells in the tumor compared to periphery, were observed, leading to anti-tumor effects in combination with anti-PD-L1. The B16.F10 model is very aggressively growing and contains only few and rather immunosuppressive cell types (“cold tumor”) [40], which might have contributed to the limited monotherapy effects seen in this model not only for GT-00AxIL-15, but also for anti-PD-L1. In contrast, the CT26.wt colon cancer model is described to be highly immune-infiltrated (“hot tumor”) and sensitive to treatment with anti-PD-L1 [40]. In this model, GT-00AxIL15 monotherapy significantly delayed tumor growth comparable to anti-PD-L1 treatment. To circumvent immunogenicity against the human construct in immunocompetent mice potentially limiting therapeutic effects, a PBMC-humanized prostate cancer model was also investigated. GT-00AxIL15 significantly reduced TV in the challenge setting, but even increased TV in the therapeutic setting, which was analytically confirmed as pseudo-progression due to infiltration with immune cells. We speculate that a longer observation period would have been necessary, as only around day 19 critical amounts of IL-15-responsive effector cells necessary for anti-tumor responses were reached, but this was not possible due to ethical reasons and limitation by onset of Graft-versus-host-disease. In contrast to what we observed for GT-00AxIL15, in vivo anti-tumor effects of other IL-15 (super)agonists on primary tumor growth are rather weak when applied as monotherapy. Effects are mainly observed in metastasis models or in combination with other therapeutics [9,15,23]. GT-00AxIL15 also synergized with approved ADCC-mediating αEGFR or αPD-L1 mAbs in vitro and induced significant anti-tumor activity in combination with αPD-L1 treatment in a melanoma model that is non- or minimally responsive to IL-15 or αPD-L1/PD-1 monotherapy [41]. These data highlight GT-00AxIL15’s potential as enhancer for other immunotherapies that stimulate distinct pathways; an approach which is already being tested with other IL-15 agonists [42], also in the clinical setting, e.g., NKTR-255 + cetuximab (NCT04616196), SOT-101 + pembrolizumab (NCT05256381) or XmAb24306 + atezolizumab (NCT04250155).

The mechanism of immune activation of GT-00AxIL15 is not strictly tumor target-dependent and do not require receptor crosslinking as e.g., for ADCC mediating mAbs or T cell engaging bispecific antibodies, which do only induce immune activation after tumor target binding. GT-00AxIL15 rather directs activated immune effector cells from the periphery and lymphoid organs towards to tumor via its TA-MUC1 binding domain. Immune activation in the periphery and lymphoid organs is actually the mechanism of action of nearly all other non-tumor-targeted IL-15 based immunocytokines, which show promising results in the clinic. This systemic activation contributes to the anti-tumoral effects, but GT-00AxIL15 adds the benefit of targeting and retention in the tumor, thereby further enhancing local IL-15 concentrations in relation to the tolerable overall exposure. Considering the participation of systemic activation in the overall mechanism of action, it should be mentioned, that in all in vivo studies, treatment with GT-00AxIL15 was well tolerated and no relevant side effects were observed. It is further noted that preclinical safety studies of GT-00AxIL15 showed a favorable safety profile (not published). For example, in a 4 week GLP toxicology study in rats, repeated doses of up to 2.5 mg/kg GT-00AxIL15 i.v. induced the expected PD effects on NK, NKT and cytotoxic T cells in blood, but did not induce test item-related side effects or signs for inflammation, like e.g., cytokine release, body or organ weight changes or histopathologic findings in organs at final necropsy.

Overall, the data support our development rationale and indicate that GT-00AxIL15 has great potential as a next-generation tumor-targeted IL-15-based immunocytokine both as monotherapy and as combination partner for other cancer therapeutics in a broad range of solid tumor indications.

## 4. Materials and Methods

### 4.1. Test and Control Articles

GT-00AxIL15, its parental anti-TA-MUC1 mAb GT-00A and an untargeted MOPCxIL15 isotype control lacking TA-MUC1 binding were generated by Glycotope as described in Supplemental methods. rh-IL15 (Miltenyi, Bergisch Gladbach, Germany), human IgG1 (Sigma (St. Louis, MO, USA) or Biolegend (San Diego, CA, USA)), αEGFR mAb cetuximab (Erbitux, Eli Lilly and Merck KGaA, Darmstadt, Germany), αPD-L1 mAb avelumab (Bavencio, Merck Serono and Pfizer) and αPD-L1 clone 10F.9G2 (ratIgG2bκ, BioXCell, Lebanon, NH, USA) were purchased from commercial sources. For in vitro binding studies using primary human cells, GT-00AxIL15 was labelled with Alexa Fluor 647 according to manufacturer´s instructions (Molecular Probes, Invitrogen, Waltham, MA, USA). For in vivo biodistribution studies, GT-00AxIL15 and MOPCxIL15 were radiolabeled with the radionuclide zirconium-89 (^89^Zr) by conjugation of p-SCN-Bn-Deferoxamine to lysine amino acid residues on the antibodies via a thiourea linkage and purified by size-exclusion chromatography. Radiopurity and specific activity was comparable for both samples (>99% and 60 MBq/mg).

### 4.2. Cells and Cell Culture

All cell culture reagents were purchased from Biowest and Serana. Tumor cell lines ZR-75-1, MCF-7, HSC-4, CaoV-3 and T-47D as well as the human NK cell line KHYG-1 and murine T cell line CTLL-2 were obtained from DSMZ or ATCC and cultured in the respective recommended medium under standard conditions. KHYG-1 and CTLL-2 were cultured in presence of 10 ng/mL IL-2 (PeproTech, Rocky Hill, NJ, USA).

For in vivo studies, the murine tumor cell lines B16.F10 and CT26.wt (ATCC) were stably transfected by electroporation (Amaxa) with a plasmid coding for human MUC1 (40 tandem repeats) and confirmed for TA-MUC1 expression in vitro and in vivo in mice by flow cytometry and immunohistochemistry using GT-00A. Cells were cultured in standard medium +100 nM methotrexate (Hexal, Holzkirchen, Germany) as selection pressure. For the metastasis model, hMUC1-B16.F10 cells were further transduced to stably express firefly luciferase (110 RLU/cell) enabling detection of metastasis. Origin, TA-MUC1 and IL-15R expression status of cell lines are indicated in Table S1.

Peripheral blood mononuclear cells (PBMCs) were prepared from commercially available buffy coats (DRK Berlin) or leukapheresates (Charité Berlin) of healthy donors by Biocoll separation (Biochrom, Cambridge, UK) and density gradient centrifugation. PBMCs from leukapheresate products were stored frozen in liquid nitrogen.

### 4.3. Flow Cytometry

Flow cytometry was used for cellular binding assays, signaling and activation assays as well as immune cell proliferation and phenotyping in vitro and in vivo as described in Supplemental methods. Table S2 summarizes all fluorochrome-labeled antibodies used for flow cytometry analysis. A viability dye (DAPI or 7-AAD) was always included to sort out dead cells. All analyses were performed on a FACS Canto II (Becton Dickinson Biosciences, Franklin Lakes, NJ, USA) or Attune NxT Flow Cytometer (Thermo Fisher Scientific, Waltham, MA, USA). Instrument settings were set by machine software and calibration beads. Data were analyzed using FlowJo 10 software (Treestar, Woodburn, OR, USA). Gates for positive markers were set according to isotype controls.

### 4.4. IL-15 Bioactivity Assay

KHYG-1 cells were harvested from continuous culture and starved from IL-2 for 4 h. Afterwards, cells were incubated for 2 days at 37 °C with GT-00AxIL15, hIgG1 or rhIL-15 and proliferation was determined by analysis of viable cells using the CellTiter-Glo Luminescent Cell Viability Assay (Promega, Madison, WI, USA) according to manufacturer’s instructions. Luminescence was recorded with a TECAN infinite 200 PRO (Tecan, Seestrasse, Switzerland).

### 4.5. Cytotoxicity Assays

Target tumor cells (ZR-75-1, MCF-7, T-47D) were loaded with europium (Eu^3+^, SIGMA) by electroporation using a Nucleofector (Amaxa). After washing, target cells were incubated with test and control substances in the presence of PBMCs as effector cells for 5 h, either in a defined effector to target cell (E:T) ratio of 80:1 or at a fixed test item concentration (20 nM) and varying E:T ratios ranging from 100:1 to 10:1. Cell lysis was analyzed by quantifying europium release in target cell supernatant at 340/630 nm with a fluorescence plate reader Infinite 200 Pro (Tecan).

### 4.6. In Vivo PK and PD Properties in Tumor-Free Mice

Female immunocompetent C57BL/6 mice were used in all studies. Test articles were administered by i.v. bolus injection into the tail vein (5–10 mL/kg) and phosphate buffered saline (PBS) served as vehicle control where indicated.

For the PK study, three mice each received a single dose of 2 mg/kg GT-00AxIL15 or molar equivalent of 0.29 mg/kg rhIL-15. Blood samples were taken 5 min, 6 h and 1, 2, 4, 7 and 11 days post-injection for analysis of GT-00AxIL15 levels in serum by enzyme-linked immunosorbent assay (ELISA) as described in Supplementary methods. Serum rhIL-15 levels were determined 5 min, 2 and 7 h post-injection by a commercial sandwich ELISA (R&D Systems, Minneapolis, MN, USA). Terminal half-life was determined in GraphPad Prism 9 (GraphPad Software, Boston, MA, USA).

To assess PD properties, three mice each received a single dose of 1 mg/kg GT-00AxIL15. Blood, spleen and inguinal lymph nodes (ingLN) were collected 0, 2, 3, 4, 5 or 8 days post-injection and processed as single cell suspensions as described in Supplemental methods. In another study comparing PD effects of different doses and single (d0) vs. multiple dosing (d0/7), six mice each received 0.1, 0.5 or 2.5 mg/kg GT-00AxIL15. Blood samples were collected on days 3, 7, 10, 14 and 21. For both in vivo PD studies, flow cytometry analysis of blood cells was performed as described in Supplemental methods to discriminate different lineages and detect their activation status.

### 4.7. Biodistribution and In Vivo Efficacy in Syngeneic Mouse Tumor Models

To investigate the biodistribution and in vivo efficacy of GT-00AxIL15, hMUC1-B16.F10 murine tumor cells were inoculated into female C57BL/6 mice. For the biodistribution study, 5 × 10^5^ hMUC1-B16.F10 tumor cells in 0.1 mL PBS were implanted s.c. into the flank. When tumors reached an average volume of 0.2 cm^3^, ^89^Zr-labeled GT-00AxIL15 and MOPCxIL15 were administered i.v. through the tail vein (5 mL/kg) at doses of 0.75 and 3 mg/kg and mice were imaged under static Positron Emission Tomography (PET) at 2, 24, 48 and 72 h post-injection (20 min under anesthesia). Animals were euthanized immediately after the final PET scan, and radioactivity in dissected tissues (as given in Figure 5A) and in blood samples drawn from the tail vein at 24, 48 and 72 h was determined in the gamma-counter. Mean percentage of injected dose per gram (%ID/g) was determined for all tissues and tumor/blood ratio was calculated.

For the hMUC1-B16.F10 melanoma metastasis model, on day 0 mice were inoculated i.v. with 2 × 10^5^ tumor cells and randomized based on body weight (bw). 12 mice each were treated with vehicle, 0.1 or 0.5 mg/kg GT-00AxIL15 i.v. on days 1 and 8. On day 15, organs were collected, mice with metastases were counted and luciferase activity was measured in the lung as main target organ for metastasis.

hMUC1-B16.F10 cells were also inoculated into the left mammary fat pad (mfp) of mice (2 × 10^5^ or 5 × 10^5^ in 100 µL PBS) on day 0 to establish solid tumors. In the first study (challenge setting), animals were randomized on day 1 based on bw and 12 animals each received vehicle or 0.5 mg/kg GT-00AxIL15 (i.v.) alone or in combination with 200 µg αPD-L1 (i.p.) on days 1, 8 and 15. Animals were euthanized on day 36 and tumor volumes (TV) were determined. In a second study, animals were randomized to groups of 8 animals each on day 8, when tumors reached a mean TV of ~130 mm^3^, and received a single i.v. injection of either vehicle, 0.1 or 0.5 mg/kg GT-00AxIL15. Three days after treatment, mice were sacrificed, and tumors and spleen were collected for flow cytometric analysis (Supplemental methods) to discriminate different lineages and to detect their activation status and proliferation. Another syngeneic (hMUC1-CT26.wt) colon cancer model is described in Supplemental methods.

### 4.8. In Vivo Efficacy in Humanized DU-145 Prostate Cancer Model (Challenge Setting)

Male NCG mice were inoculated s.c. in the flank with a mix of 5 × 10^6^ DU-145 tumor cells (PBS/Matrigel, 1:1) and 2.5 × 10^6^ human PBMCs in 0.2 mL PBS on day 0 and randomized based on bw. 20 mice each (*n* = 4 for 5 PBMC donors) received i.v. injections of vehicle, 0.5 or 0.25 mg/kg GT-00AxIL15 (10 mL/kg) on study days 1, 8 and 15. Animals were checked daily for morbidity and mortality. Bw and TV were monitored 3 times per week. Animals with tumors of a TV > 1.5 cm^3^ or a bw loss over 20% were euthanized for ethical reasons.

### 4.9. Statistical Analyses

Statistical analyses of all quantitative data were performed using GraphPad Prism Version 5.01 for Windows (GraphPad Software) and Microsoft Excel (Microsoft, Redmond, WA, USA). For comparison between more than two groups analysis of variance (ANOVA) was performed with post-hoc tests as indicated in figure legends

## Figures and Tables

**Table 1:** Anti-tumor activity of GT-00AxIL15 in humanized prostate cancer model.

PBMC Donor	TGI [%] by GT-00AxIL15		
	Day	2.5 mg/kg	0.5 mg/kg
A	26	57.7	5.9
B	32	92.5	−10.9
C	31	62.9	25.6
D	19	0.9	−8.3
E	32	68.7	−24.6

TGI = Tumor Growth Inhibition: TGI% = (1 − Ti/Vi) × 100; Ti as the mean TV of the treatment group on the day of measurement; Vi as the mean TV of control group on the day of measurement. Re-sults from *n* = 4 mice/donor and treatment.

## Data Availability

Data are available on reasonable request.

## Supplementary Materials

The following supporting information can be downloaded at: https://www.mdpi.com/article/10.3390/ijms25031406/s1. Reference [43] is cited in the supplementary materials.
