# Supplementary material for: GT-00AxIL15, a Novel Tumor-Targeted IL-15-Based Immunocytokine for the Treatment of TA-MUC1-Positive Solid Tumors: Preclinical In Vitro and In Vivo Pharmacodynamics and Biodistribution Studies
Source: Int J Mol Sci. 2024 Jan 24;25(3):1406. doi: 10.3390/ijms25031406 (PMC10855649; doi:10.3390/ijms25031406)
Supplement: Supplementary file 1 [file ijms-25-01406-s001.zip › ijms-2807728-supplementary.pdf]

Supplementary Material

GT-00AxIL15, a novel tumor-targeted IL-15-based immunocytokine for the treatment of TA-MUC1-positive solid tumors: preclinical in vitro and in vivo pharmacodynamics and biodistribution studies

Johanna Gellert*, Anika Jäkel, Antje Danielczyk, Christoph Goletz, Timo Lischke, Anke Flechner, Laura Dix, Alexandra Günzl , Patrik Kehler

*** Correspondence:**
Johanna Gellert
Johanna.gellert@glycotope.com

1 **Supplementary Methods** 3
1.1 Generation of GT-00AxIL15 and the controls MOPCxIL15 and GT-00A 3
1.2 Specificity analysis by Enzyme-Linked Immunosorbent Assay (ELISA) 3
1.3 Binding kinetics and affinity for TA-MUC1 antigen 4
1.4 Fcγ receptor Alpha Screen 4
1.5 Cellular binding to TA-MUC1 and IL-15R 4
1.6 IL-15R downstream-signaling in vitro 4
1.7 Immune cell proliferation assay in vitro 5
1.8 Immunophenotyping of effector cells in vitro 5
1.9 In vitro 3D co-culture assay of PBMCs with TA-MUC1^{+} tumor spheroids 5
1.10 In vitro combination cytotoxicity assays 5
1.11 Quantification of GT-00AxIL15 for PK evaluation 6
1.12 Flow cytometry to investigate PD effects in blood, lymphoid organs and tumors in vivo 6
1.13 In vivo efficacy of GT-00AxIL15 in syngeneic hMUC1-CT26.wt colon cancer model 7
1.14 In vivo efficacy in humanized mouse model – therapeutic setting 7
2 **Supplementary Figures** 8
Supplementary Figure S1. GT-00AxIL15 binds to its targets TA-MUC1, IL15R and FcyR in vitro. 8
Supplementary Figure S2. GT-00AxIL15 activates IL-15R downstream-signaling in vitro 9
Supplementary Figure S3. GT-00AxIL15 induces infiltration of T cells into 3D tumor spheroids in vitro. 9
Supplementary Figure S4. GT-00AxIL15 acts synergistically with ADCC-mediating mAbs in vitro 10
Supplementary Figure S5. GT-00AxIL15 induces activation and proliferation of immune effector cells in vivo with comparable kinetics after single and repeated dosing 11

Supplementary Figure S6. GT-00AxIL15 shows in vivo efficacy in the syngeneic hMUC1-CT26.wt colon cancer model (challenge setting) 12
Supplementary Figure S7. GT-00AxIL15 elicits pharmacodynamic effects in humanized DU-145 prostate cancer model (therapeutic setting) 13
3 Supplementary Tables 14
Supplementary Table S1: Cell lines used 14
Supplementary Table S2. FACS antibodies used 15
Supplementary Table S3. TA-MUC1 binding kinetics of GT-00AxIL15 17
Supplementary Table S4. Immunophenotyping of PBMCs from tumor cell co-cultures after treatment with GT-00AxIL15 in vitro 17
Supplementary Table S5. Pharmacodynamic effects of GT-00AxIL15 on peripheral versus tumor immune cell infiltrates in vivo 18

1 Supplementary Methods

1.1 *Generation of GT-00AxIL15 and the controls MOPCxIL15 and GT-00A*

The variable regions of GT-00AxIL15 are based on the sequence of gatipotuzumab (GT-00A, humanized hIgG1 PankoMab (PM) produced in GEX cells), but includes the N54Q mutation in complementary-determining region 2 of the heavy chain (HCDR2) to remove its Fab N-glycosylation. The antibody sequences of the variable heavy (VH) and light (VL) region were cloned into expression vectors containing sequences for the human constant domains of the IgG1 κ light chain and heavy chain (Glycotope), whereas the heavy chain plasmid additionally contains the human IL-15 sequence fused C-terminally *via* the rigid linker sequence $(PAPAP)_6$. The N-glycosylation site at position N297 relevant for Fc functionality is preserved in GT-00AxIL15 and C-terminal lysine of the IgG heavy chain is mutated to alanine to prevent cleavage by proteases (K447A) (see Figure 1A). Both plasmids were co-transfected in host cells, whereas different expression systems were used during development: *In vitro* studies used GT-00AxIL15 material produced under R&D conditions in the human myelogenous cell line GEX (Glycotope). For *in vivo* studies and large-scale production, Chinese hamster ovary cells (CHO) were used. Producing cells were selected by methotrexate (Sigma-Aldrich) and for GEX cells also with puromycin (Clontech) and single clones were generated, expanded and used for production of supernatants in bioreactors. GT-00AxIL15 was purified by protein A chromatography and at least one polishing step to a purity ≥98%. Comparability of both materials from GEX and CHO was confirmed in both *in vitro* pharmacology and *in vivo* pharmacokinetics (data not shown, but available upon request).

As controls, an irrelevant isotype control lacking TA-MUC1 binding but including the same Fc part and IL-15 modules (MOPCxIL15) was generated as described above under R&D conditions in GEX. Here, the variable sequences of mouse IgG1 MOPC-21 clone were used[1]. To compare against the parental antibody GT-00A, GMP-grade material was used (Glycotope).

All test samples were formulated in aqueous buffer, near-neutral pH.

1.2 *Specificity analysis by Enzyme-Linked Immunosorbent Assay (ELISA)*

To test binding of GT-00AxIL15 to its different targets, sandwich ELISA experiments were performed. Either synthetic biotinylated MUC1-derived peptides (Btn-Ahx-APPAHGVTSAPD-T(with or without GalNAc=Tn)-RPAPGSTAPPAHGVTSA-sre, Biosyntan) or Fc chimeras of human IL-15R subunit α/CD215 (R&D systems) or β/CD122 (abcam) were immobilized at 0.5 - 1 µg/mL on streptavidin-coated (Biotez) or Maxisorp (Nunc) 96-well plates in PBS overnight at 4 °C. After washing and blocking, GT-00AxIL15, GT-00A and MOPCxIL15 were added as primary antibodies at the indicated concentrations diluted in PBS + 1% BSA for 2 h at room temperature (RT). After washing, samples were incubated for 1 h at RT with rabbit-anti-human IgG-HRP (Jackson Immuno Research) as secondary antibody.

Further, a dual binding ELISA was performed by coating hIL15Rα/CD215-Fc followed by washing, blocking and incubation with serial dilutions of GT-00AxIL15 or controls. After washing, antibodies with ability to bind both targets simultaneously were detected *via* the biotinylated Tn-glycosylated MUC1 peptide and POD-Streptavidin (Jackson Immuno Research).

For all ELISAs, substrate reaction was performed with 3,3',5,5'-tetramethylbenzidine (TMB; TEBU GmbH) and after stopping with 2.5 N H_2SO_4, absorbance was measured at 450 nm by multimode microplate reader (PerkinElmer or Tecan).

---

[1] Sibinovic et al.: Catalogue of plasmacytomas and other tumors of the lymphoreticular system, 3rd edition. Kensington, Maryland: Litton Bionetics, Inc; 1976:1-33

1.3 Binding kinetics and affinity for TA-MUC1 antigen

The affinity of GT-00AxIL15 to its tumor antigen TA-MUC1 was determined by the switch sense technology (proximity sensing, i.e., modification of a DNA-ligated fluorophore) on a DRX^2 instrument (Dynamic Biosensors). First, the nanolever chip channel was passivated with passivation solution as recommended by the manufacturer. The nanolever cNL-B96 modified with Streptavidin (200 nM) was mixed with an equal volume of non- modified cNL-A96 (200 nM) and diluted fourfold with PE40 buffer. The mix was applied to the chip in each cycle for 20 min at a flow of 1.25 µL/min. The biotinylated TA-MUC1 peptide (Btn-Ahx-(HGVTSAPD-T(GalNAc)-RPAPGSTAPPA)$_3$-OH x TFA, Biosyntan) was diluted in PE140 to 200 nM (1.33 µg/mL). It was then applied to the streptavidin-coated chip for 75 s with a flow of 100 µL/min. The analyte GT-00AxIL15 was diluted in PE140 to 100, 11 and 1.23 nM and applied in subsequent cycles for 60 s at 50 µL/min and dissociated for 10 min at 200 µL/min. The measurement mode was proximity sensing, which means that the fluorescence signal of the nanolever labels is monitored. The binding curves of association and dissociation were evaluated with the DRX^2 method "Global Fit, Mono-Exponential". The chip was regenerated after each cycle with regeneration solution in the "stand by" master. The equilibrium dissociation constant K_D was calculated from the ratio of k_{off}/k_{on}.

1.4 Fcγ receptor Alpha Screen

To determine binding of the Fc moiety of GT-00AxIL15 and controls to FcγRIIIa, binding assays using the AlphaScreen technology (bead-based technology) of PerkinElmer were performed. His-tagged FcγRIIIa (Glycotope) diluted in 1x AlphaLisa Universal Buffer (PerkinElmer) and test antibodies GT-00AxIL15, MOPCxIL15 and GT-00A were pre-incubated for 30 min at RT. The pre-incubated antibody-receptor mix was incubated with a 25 µg/mL mix of donor (AlphaScreen nickel chelate donor beads, Perkin Elmer) and acceptor (AlphaScreen rabbit-anti-mouse acceptor beads, Perkin Elmer) beads in 1x AlphaLisa Universal Buffer for 1 h at RT in the dark. His-tagged FcγRIIIα was captured by donor beads. Test samples and the rabbit antibody coupled to the acceptor beads compete for binding to FcγRIIIα. The close proximity of donor and acceptor beads leads to light emission in the acceptor bead. A maximum signal is achieved without a competitor; in case of competition, where a rabbit antibody binds to FcγRIIIa with the acceptor beads the maximum signal is reduced in a concentration-dependent manner. After laser excitation at 680 nm chemiluminescence was quantified by measurement at 520-620 nm (AlphaScreen method) with an EnSpire 2300 multilabel reader (PerkinElmer).

1.5 Cellular binding to TA-MUC1 and IL-15R

To test binding to cellular TA-MUC1 on ZR-75-1 and MCF-7 or IL-15R on murine CTLL-2 cells, $1x10^5$ cells/well were incubated with GT-00AxIL15, MOPCxIL15 or GT-00A diluted to indicated concentrations in PBS/0.2% BSA (30 min/4 °C/dark). Bound antibodies were detected by flow cytometry with PE-conjugated anti-human IgG-Fc secondary antibody (Jackson).

Binding to IL-15R on primary human immune cells was investigated by incubating $2x10^5$ PBMCs from 3 healthy donors with serial dilutions of Alexa Fluor 647-labeled GT-00AxIL15 (30 min/RT/dark) followed by incubation with test antibody and fluorochrome-labeled antibodies to discriminate immune cell subsets (NK, NKT, $CD4^+$ and $CD8^+$ T cells) (30 min/RT/dark). To avoid binding to Fcγ receptors, PBMCs were pre-incubated (10 min/RT/dark) with 2 mg/mL normal human immunoglobulin Octagam (Octapharma) diluted in PBS/0.2% BSA.

1.6 IL-15R downstream-signaling in vitro

To characterize IL-15-mediated signal transduction, flow cytometry was used to determine the phosphorylation status of Signal transducer and activator of transcription 5 (STAT5) in immune cell populations with the mAb clone 47/STAT5(pY694) that specifically recognizes STAT5 when

phosphorylated at Y694. PBMCs from four healthy donors (5x10^5 cells/well) were stimulated for 20 min at 37 °C with serial dilutions of GT-00AxIL15, GT-00A or rhIL-15 or left untreated. Cells were washed, fixed with 10% formalin (Sigma Aldrich) and permeabilized with Phosflow PermBuffer III (BD Biosciences) to be analyzed for intracellular pSTAT5 expression in CD3$^-$ CD56$^+$ NK cells, CD3$^+$ CD8$^+$ and CD3$^+$ CD8$^-$ T cells as well as in CD56$^-$ CD3$^-$ lymphocytes (mainly B cells) by flow cytometry.

1.7 *Immune cell proliferation assay in vitro*

PBMCs from healthy human donors were labeled with 2 μM CellTrace Violet (CTV, Thermo Fisher) at 1x10^6 cells/mL in PBS at 37 °C for 20 min. Afterwards, 2x10^5 cells/well were incubated with GT-00AxIL15 or controls at indicated concentrations for 5 days at 37 °C. The proliferation of NK, NKT, CD4$^+$ and CD8$^+$ T cells was assessed by the percentage of the divided cell population as measured by CTV dilution using flow cytometry.

1.8 *Immunophenotyping of effector cells in vitro*

To assess the phenotypic changes induced by GT-00AxIL15 in immune effector cells, expression levels of different cell surface markers were analyzed by flow cytometry. Healthy donor PBMCs (2x10^5 cells/well) were incubated with 2x10^4 CaoV-3 tumor cells with or without 20 nM GT-00AxIL15 for 3 days at 37 °C followed by detection with fluorochrome-labeled antibodies (see Table S2) for 30 min at 4 °C. Cell surface expression of investigated molecules was determined by % positive cells or change in median fluorescence intensities in case of 100% cells stained positive.

1.9 *In vitro 3D co-culture assay of PBMCs with TA-MUC1+ tumor spheroids*

MCF-7 breast cancer cells (1.6x10^3 cells/well) were grown for three days in 96-well spheroid microplates at 37 °C. Test items (GT-00AxIL15, MOPCxIL15, GT-00A) were added at a concentration of 57 nM, medium served as untreated control. After 4 hours, spheroids were washed and 1x10^5 PBMC from a healthy donor were added. After 48 hours, spheroids were harvested, embedded in paraffin and slides were subjected to immunohistochemistry analysis to determine the amount of infiltrated T cells. After deparaffinization and rehydration in a descending alcohol series and antigen unmasking by high temperature (95 °C) treatment, slides were stained with rabbit anti-human CD3 (clone SP7, abcam) or mouse anti-human CD8a (clone C8/144B; BioLegend). Bound antibodies were detected by peroxidase conjugated goat anti-rabbit IgG-(H+L) (Jackson Immuno Research) or Envision Flex anti-mouse Ig-HRP (Dako) and Liquid DAB+ Substrate Chromogen System (Dako). Slides were counterstained with Mayer's Hematoxylin (Thermo Fisher), incubated in ascending alcohol series and mounted in Entellan (Merck). Slides were analyzed by microscopy (Axioplan 2 Imaging) and infiltrated immune cells per individual spheroid were determined using the ZEN software (ZEISS).

1.10 *In vitro combination cytotoxicity assays*

Tumor cell killing of GT-00AxIL15 was assessed in combination with other ADCC-mediating anti-tumor antibodies. Highly immunosuppressive and TA-MUC1-, EGFR- and PD-L1-expressing HSC-4 target tumor cells (1.5x10^4 cells/well) were seeded overnight at 37 °C in 96-well plates. On the next day, human primary PBMCs were added (2x10^5 cells/well corresponding to an E:T ratio of ~10:1) and incubated for 24-48 h with GT-00AxIL15 and anti-EGFR mAb cetuximab (Erbitux), anti-PD-L1 avelumab (Bavencio) or hIgG1 isotype control at indicated concentrations. Tumor cell lysis was assessed by quantification of lactate dehydrogenase (LDH) released into cell supernatant (Cytotoxicity Detection Kit (LDH), Roche) by measurement at 490 nm by multimode microplate reader (PerkinElmer).

1.11 *Quantification of GT-00AxIL15 for PK evaluation*

Serum samples from the mouse PK study were analyzed for GT-00AxIL15 titer by sandwich ELISA. Maxisorp plates (Nunc) were coated overnight at 4 °C with 2 µg/mL mouse-anti-human Igk light chain antibody (BD Pharmingen). After washing and blocking with 2% BSA/PBS, serum samples and a GT-00AxIL15 calibration curve were added in duplicates, diluted in 1% BSA/PBS containing 5% mouse serum, as the minimum required dilution was determined to be 1:20. After 1 hour incubation at RT, plates were washed and bound antibodies were detected by goat-anti-human IgG Fc-POD secondary antibody (Jackson Immuno Research). Substrate reaction was performed with 3,3′,5,5′-tetramethylbenzidine (TMB; TEBU GmbH) and after stopping with 1.5 N H_2SO_4, absorbance was measured at 450 nm by multimode microplate reader (Tecan). Serum titers were back-calculated from the calibration curve and each run was controlled by system suitability testing using quality control (QC) samples at the upper and lower assay range with an admissible recovery of 70-130%.

1.12 *Flow cytometry to investigate PD effects in blood, lymphoid organs and tumors in vivo*

To investigate peripheral versus tumor PD effects in mice in vivo, blood, tumors, lymph nodes and spleens harvested from mice subjected to in vivo experiments were prepared for flow cytometric analyses as follows: in defined volumes of whole blood samples erythrocytes were lysed using ACK lysing buffer (155 mM NH_4Cl, 10 mM $KHCO_3$, 100 µM EDTA [pH ~7.2]) incubation and remaining PBMCs were stained with fluorophore-coupled antibody conjugates for flow cytometric analyses. Disintegrated tumors were enzymatically digested using the mouse tumor dissociation kit (Miltenyi, #130-096-730) and then disrupted in a gentleMACS dissociator (Miltenyi) to obtain tumor cell suspensions. Disintegrated lymph nodes and spleens as well as tumor cell suspensions were mashed through 70 µm pore nylon cell sieves in PBS twice to obtain cell suspensions, only the splenocyte suspensions were subsequently subjected to ACK buffer lysis. All cells isolated from solid organs were resuspended in medium (RPMI1640 + 10% FCS + 0.05 mM β-mercaptoethanol) and cells in suspensions were counted after staining with Guava ViaCount reagent (Luminex, #4000-0041) at a Guava EasyCyte 10HT cytometer (Luminex) using the ViaCount software module. Cells isolated from solid organs were stained with fluorophore-coupled antibody conjugates for flow cytometric analyses. DAPI (0.3 µM, Merck, #124653) was added to live cell samples prior to acquisition to allow for $DAPI^+$ dead cell exclusion. For intracellular stainings, live cells were stained with 1.34 mM Pacific Orange succinimidyl ester (Thermo Fisher Scientific, #P30253) to allow for $PacO^+$ dead cell exclusion, and then fixed with eBioscience Foxp3 fixation/permeabilization buffer (Thermo Fisher Scientific, #00-5523-00). Fixed cells were then stained with fluorophore-coupled antibody conjugates in eBioscience Foxp3 permeabilization buffer (Thermo Fisher Scientific, #00-5523-00) to achieve Foxp3, Ki-67 or GzmB intracellular staining. Stained cells were acquired on a CantoII (BD Biosciences) or an Attune NxT (Thermo Fisher Scientific) flow cytometer and data were analyzed with FlowJo software (Treestar / BD Biosciences). Debris, doublets, and $DAPI^+$ or $PacO^+$ dead cells were excluded from analysis.

Quantification of specific subpopulations in lymph nodes and spleen was achieved *via* calculating total live cell counts per individual organ based on Guava EasyCyte 10HT cell count data and total volume of the organ cell suspension followed by multiplying total live cell counts with the relative frequencies of specific cell subpopulations of all live cells present in a flow cytometry sample. As cell counting with Guava EasyCyte 10HT or total live cell count data were methodically not robust enough for tumor samples, flow cytometry of tumor cell samples was applied by normalization with defined numbers of CountBright Beads (Thermo, #C36950) as internal standard to allow for quantification of tumor-infiltrating lymphocytes (TILs) in tumor cell suspensions relative to the bead count.

1.13 *In vivo efficacy of GT-00AxIL15 in syngeneic hMUC1-CT26.wt colon cancer model*

To evaluate the in vivo efficacy of GT-00AxIL15 in another syngeneic mouse model reported to be sensitive to treatment with αPD-L1, Balb/c mice were s.c. inoculated with $1x10^6$ hMUC1-CT26.wt cells in 100 µL PBS into the left flank on study day 0. On days 1, 8 and 15, 12 animals each were s.c. administered with vehicle or 0.1 mg/kg GT-00AxIL15 (at 5 mL/kg), 200 µg/mouse αPD-L1 antibody (i.p.) or a combination of 0.1 mg/kg s.c. GT-00AxIL15 and 200 µg/mouse αPD-L1 i.p.. Body weight and tumor volume (TV) were monitored 3 times per week until necropsy (day 45). Survival was assessed; animals with tumors of a TV >1.5 cm^3 were sacrificed for ethical reasons.

1.14 *In vivo efficacy in humanized mouse model – therapeutic setting*

Male NCG mice were inoculated s.c. in the right upper flank region with $5x10^6$ DU-145 tumor cells in 0.1 ml PBS/Matrigel (1:1). When tumor volumes reached ~100 mm^3, $5x10^6$ PBMCs in 0.1 ml PBS were injected i.p.; randomization was performed prior to PBMC injection. The day of PBMC injection and randomization was denoted as day 0. One day after PBMC humanization (day 1), 20 mice each (n=4 for 5 PBMC donors) received i.v. injections of PBS, 0.5 and 0.25 mg/kg GT-00AxIL15 (10 mL/kg) on study days 1, 8 and 15. Animals were checked daily for morbidity and mortality. Body weight and tumor volume was monitored 3 times per week. Animals with tumors of a TV >1.5 cm^3 or a body weight loss over 20% were euthanized. To investigate infiltration and recruitment of various immune cells, blood and tumor samples from donors B and C were collected at the end of the study (d29) for flow cytometric analysis. All samples were processed as single cell suspensions and stained with fluorophore-coupled antibodies to discriminate between murine $CD45^+$ cells and human immune cell subsets.

2 Supplementary Figures

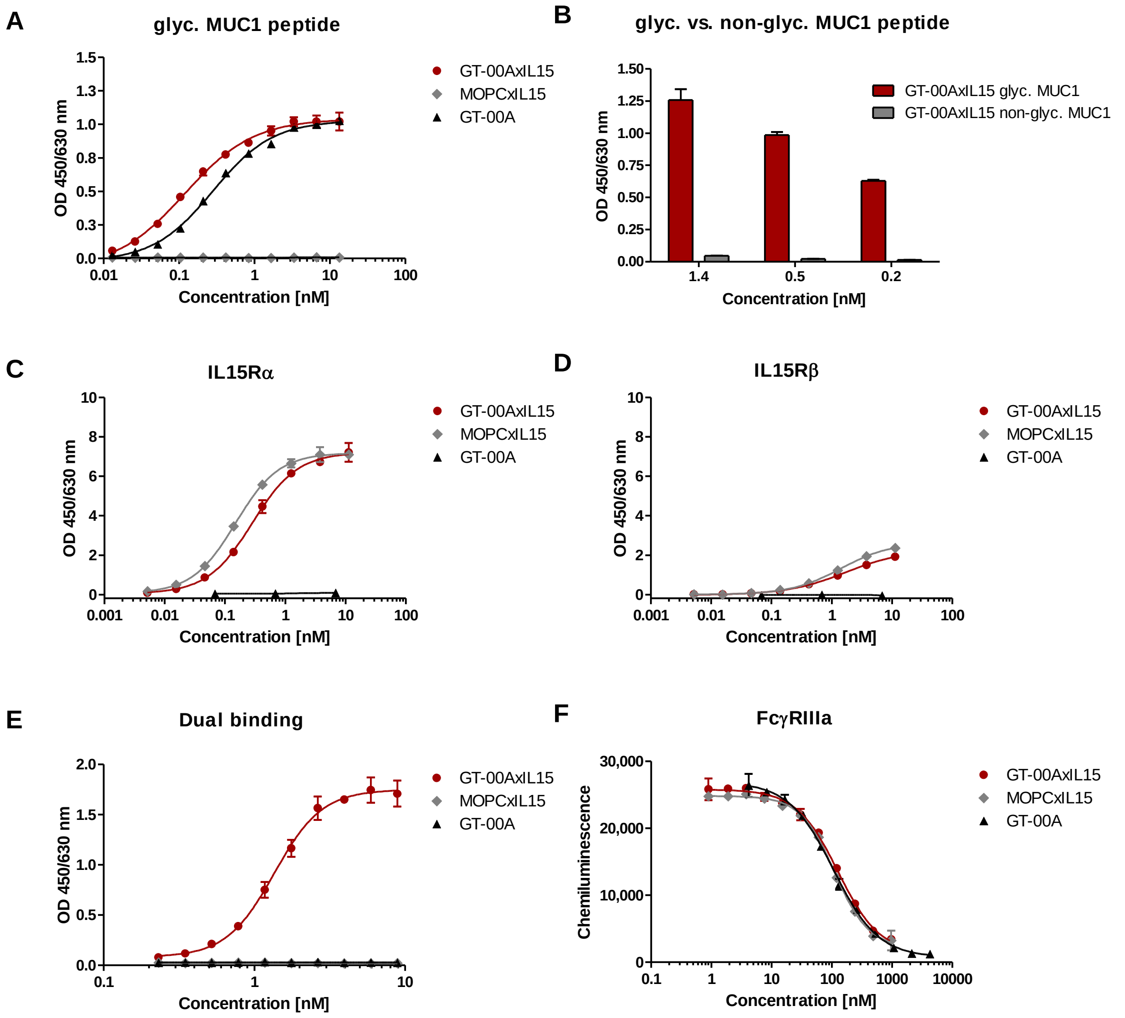

Supplementary Figure S1. GT-00AxIL15 binds to its targets TA-MUC1, IL15R and FcyR *in vitro*. ELISA binding studies with GT-00AxIL15 and controls to differently glycosylated MUC1 peptides (**A+B**), IL-15R*α* (C) and IL-15Rβ (**D**) subunit. (**E**) Dual binding to TA-MUC1 and IL-15R*α* in ELISA. (**F**) Binding to FcyRIIIa analyzed by AlphaScreen. Mean ± SD (*n* = 2) from representative experiments.

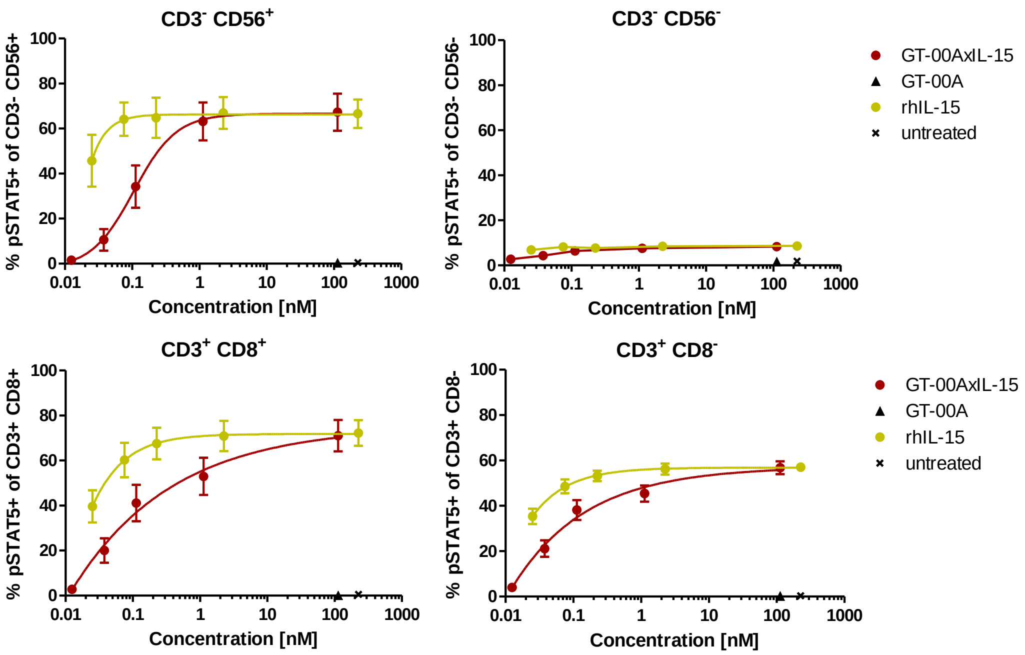

Supplementary Figure S2. GT-00AxIL15 activates IL-15R downstream-signaling *in vitro*. PBMCs were stimulated for 20 min with GT-00AxIL15, GT-00A, rhIL-15 or were left untreated. Cells were fixed, permeabilized and analyzed for intracellular pSTAT5 expression in different lymphocyte subsets by flow cytometry (from left to right: CD3- CD56+ NK cells, CD56- CD3- lymphocytes, i.e., mainly B cells, CD3+ CD8+ T cells, CD3+ CD8- T cells). Mean ± SEM from four healthy PBMC donors is given.

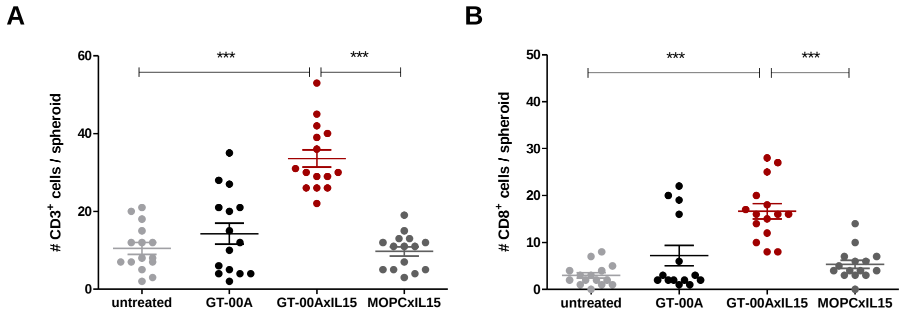

Supplementary Figure S3. GT-00AxIL15 induces infiltration of T cells into 3D tumor spheroids *in vitro*. MCF-7 tumor spheroids were treated with 57 nM GT-00AxIL15 or controls for 4 h before washing and addition of healthy donor PBMCs for further 48 h. The amount of infiltrated CD3+ (**A**) or CD8+ (**B**) immune cells was analyzed by immunohistochemistry of formalin-fixed paraffin-embedded (FFPE) slides. Mean ± SD from individual spheroid counting, one-way ANOVA, Bonferroni post-test, * $p<0.05$, ** $p<0.01$, *** $p<0.001$.

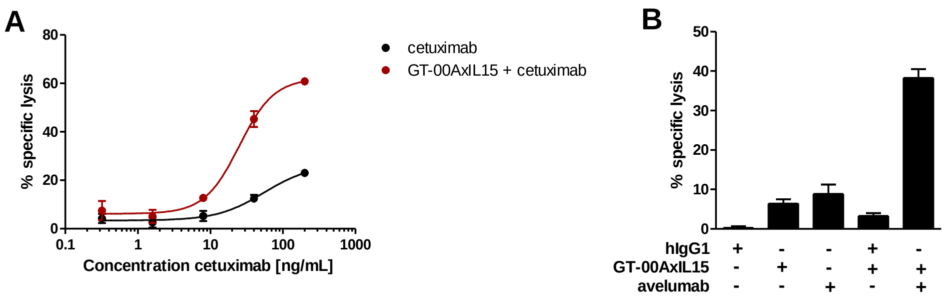

Supplementary Figure S4. GT-00AxIL15 acts synergistically with ADCC-mediating mAbs *in vitro*. ADCC assays with TA-MUC1+ EGFR+ HSC-4 tumor cells and primary human PBMCs (E:T ~10:1) after incubation with GT-00AxIL15 (**A**) at 1 nM in combination with increasing concentrations of anti-EGFR cetuximab or (**B**) at 22 nM in combination with 10 µg/mL anti-PD-L1 avelumab or hIgG1 isotype control; mean ± SD (*n*=3) from representative experiments.

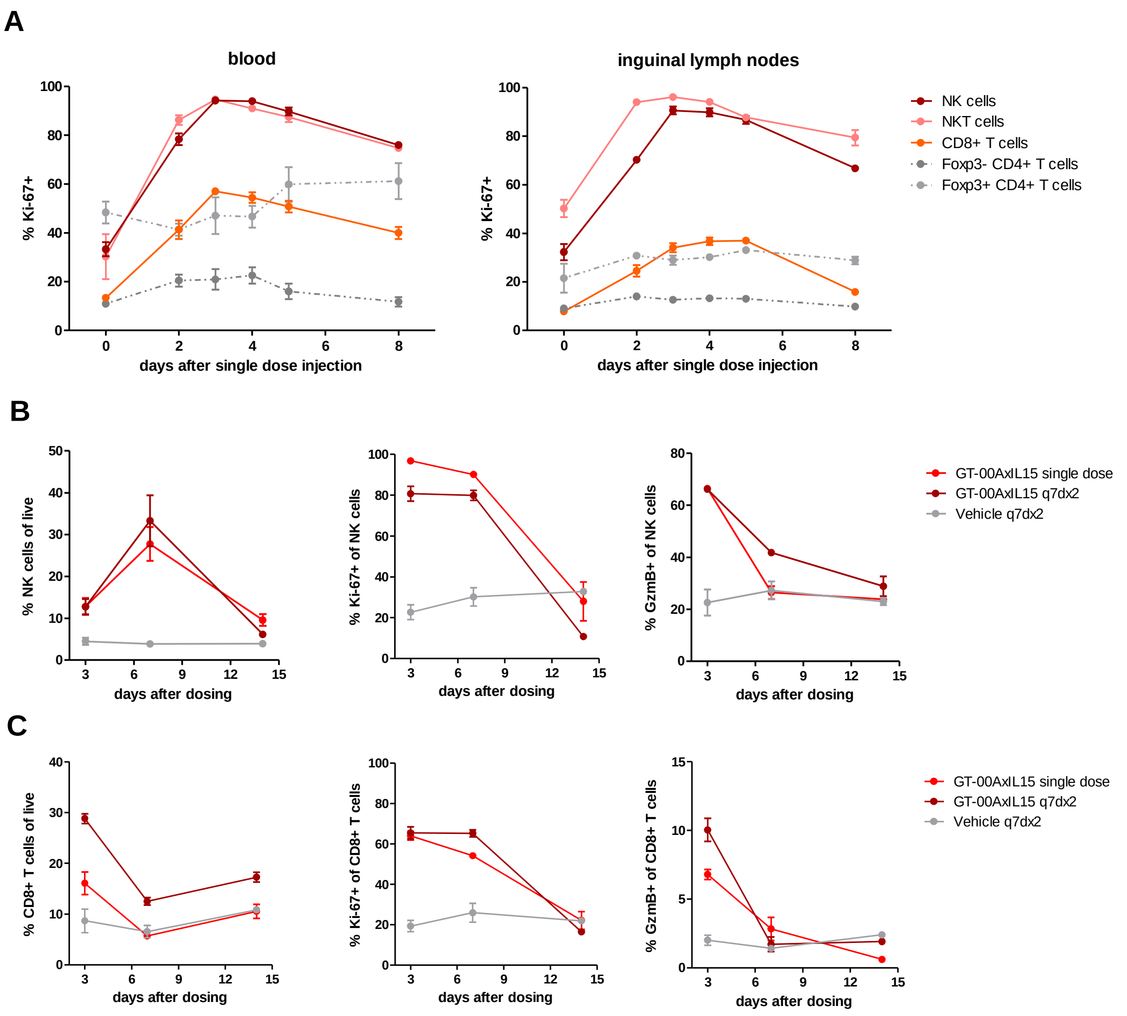

Supplementary Figure S5. GT-00AxIL15 induces activation and proliferation of immune effector cells *in vivo* with comparable kinetics after single and repeated dosing. PD effects of GT-00AxIL15 in tumor-free mice: (**A**) Relative proportion of Ki-67+ cells in NK, NKT, CD8+, Foxp3-CD4+ and Foxp3+CD4+ T-cells in blood and inguinal lymph nodes after single i.v. injection of 1 mg/kg GT-00AxIL15. (**B+C**) PD kinetics after single and multiple dosing (q7dx2) with 2.5 mg/kg GT-00AxIL15: relative proportions (left), Ki-67 (middle) and GzmB (right) expression on (**B**) NK and (**C**) CD8+ T cells. Mean ± SEM from n=3 mice.

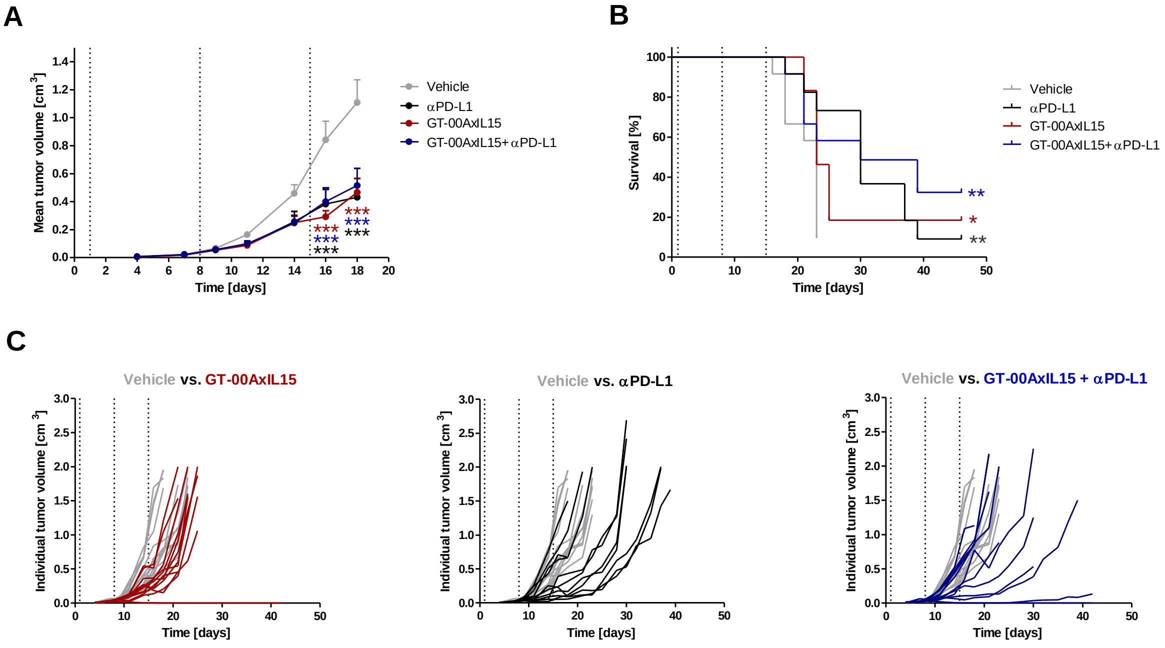

Supplementary Figure S6. GT-00AxIL15 shows in vivo efficacy in the syngeneic hMUC1-CT26.wt colon cancer model (challenge setting). Mice were implanted s.c. with hMUC1-CT26.wt tumor cells on day 0 and treated with GT-00AxIL15 (0.1 mg/kg i.v.), anti-PD-L1 (200 µg i.p.) or the combination thereof on day 1, 8 and 15 (dotted vertical lines). (**A**) Mean tumor volume ± SEM until day 18 with results from 2-way ANOVA, Bonferroni post-test, (**B**) survival curves with results from log rank test and (**C**) spider plots from n=12 mice per group are shown. * p<0.05, ** p<0.01, *** p<0.001.

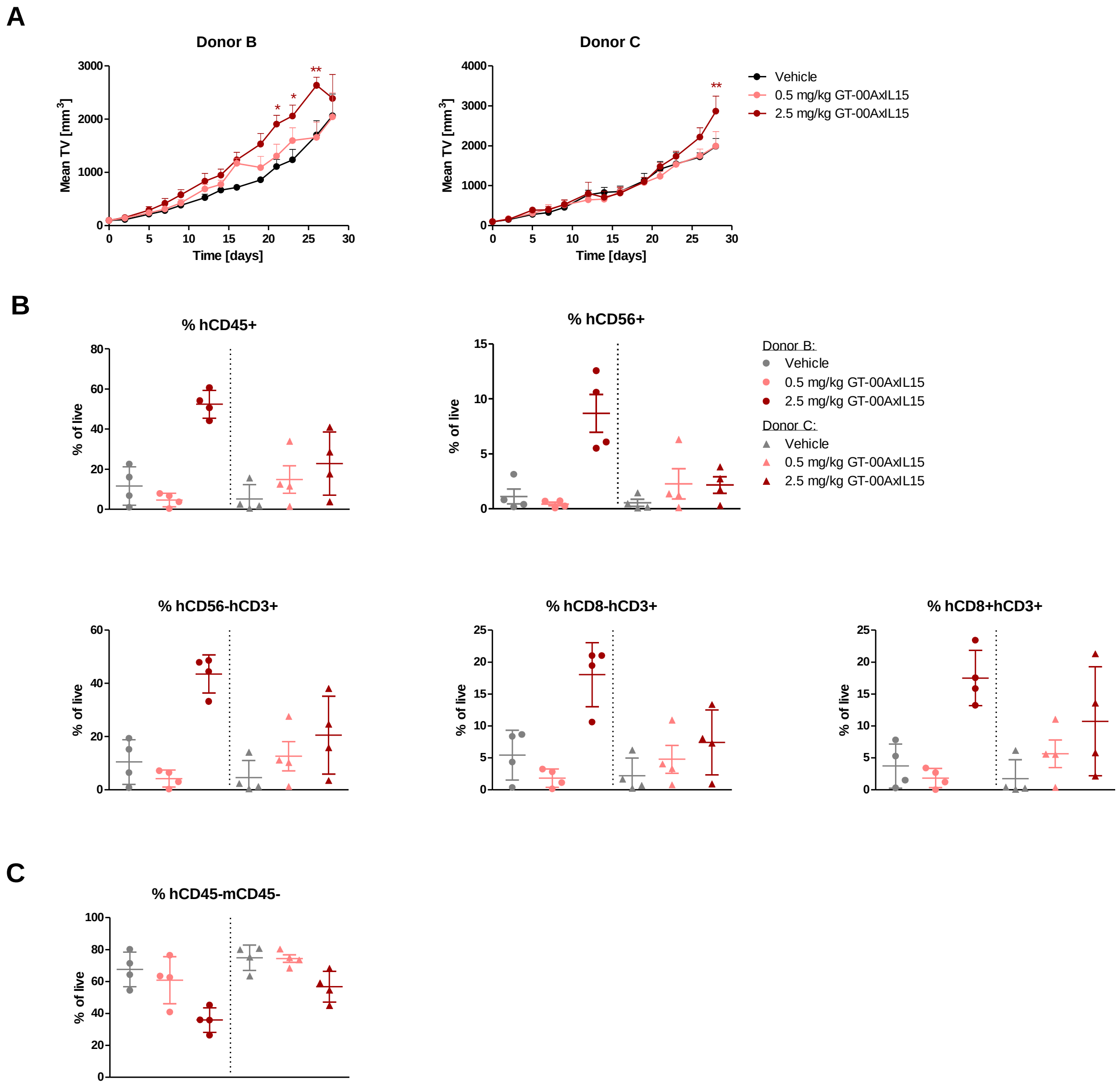

Supplementary Figure S7. GT-00AxIL15 elicits pharmacodynamic effects in humanized DU-145 prostate cancer model (therapeutic setting). Male NCG mice were s.c. inoculated with 5x10^6 DU-145 tumor cells. When tumor volume reached ~100 mm^3, 5x10^6 PBMCs were injected i.p. Treatment started one day after PBMC humanization (day 1). (**A**) Mean tumor volumes ± SEM (n=4 per individual Donor B and C); 2-way ANOVA, Bonferroni post-test, * p<0.05, ** p<0.01. (**B**) Relative proportions of human immune cell subsets in tumor on day 29. (**C**) Relative proportion of hCD45$^-$mCD45$^-$ cells reflecting mostly tumor cells. Mean ± SEM (n=4).

3 Supplementary Tables

Supplementary Table S1: Cell lines used

Cell Line	Origin	TA-MUC1	IL-15R
**Human cell lines**			
ZR-75-1	Breast	+	-
MCF-7	Breast	+	-
T-47D	Breast	+	-
CaoV-3	Ovaries	+	+
HSC-4	Tongue	+	+
DU-145	Prostate	+	+
KHYG-1	NK cells	-	+
**Murine cell lines**			
CTLL-2	T cell	-	+
hMUC1-B16.F10	Melanoma	+ (>95%)	-
hMUC1-CT26.wt	Colon	+ (20-60%)	-

Supplementary Table S2. FACS antibodies used

Assay	Reactivity + Target	Clone	Isotype	Fluorophore	Supplier
Cellular TA-MUC1 & IL-15R binding *in vitro*	hIgG(H+L)	polyclonal	goat F(ab)2	PE	Jackson ImmunoResearch
Cellular IL-15R binding *in vitro*	hCD3	OKT3	mIgG2aκ	Pacific Blue	BioLegend
	hCD4	REA623	hIgG1	PE-Vio770	Miltenyi Biotec
	hCD8a	HIT8a	mIgG1κ	PE	BioLegend
	hCD14	HCD14	mIgG1κ	APC-cyanine7	BioLegend
	hCD19	HIB19	mIgG1κ	FITC	BioLegend
	hCD56	NCAM16.2	mIgG2bκ	BV711	Becton Dickinson
Activation and proliferation of PBMCs *in vitro*	hCD3	BW264/56	mIgG2aκ	FITC	Miltenyi Biotec
	hCD4	REA623	hIgG1	PE-Vio770	Miltenyi Biotec
	hCD8	SK1	mIgG1κ	Alexa Fluor 700	BioLegend
	hCD14	HCD14	mIgG1κ	APC-cyanine7	BioLegend
	hCD45	HI30	mIgG1κ	Pacific Blue	BioLegend
	hCD56	NCAM16.2	mIgG2bκ	BV711	Becton Dickinson
	hCD25	BC96	mIgG1κ	PE	BioLegend
Immuno-phenotyping of PBMCs *in vitro*	hCD3	OKT3	mIgG2aκ	APC-Cy7	BioLegend
	hCD4	OKT4	mIgG2bκ	APC-Cy7	BioLegend
	hCD4	RPA-T4	mIgG1κ	BV421	BioLegend
	hCD8	SK1	mIgG1κ	Alexa Fluor 647	BioLegend
	hCD8	SK1	mIgG1κ	BV510	BioLegend
	hCD14	HCD14	mIgG1κ	PerCP	BioLegend
	hCD19	HIB19	mIgG1κ	PerCP	BioLegend
	hCD56	HCD56	mIgG1κ	PerCP	BioLegend
	hCD56	HCD56	mIgG1κ	PE	BioLegend
	hCD56	REA196	hIgG1	FITC	Miltenyi Biotec
	hCD25	M-A251	mIgG1κ	PE-Cy7	Becton Dickinson
	hOX-40	Ber-ACT35	mIgG1κ	PE	BioLegend
	hICOS	REA192	hIgG1	PE-Vio770	Miltenyi
	h4-1BB	4B4-1	mIgG1κ	PE	Becton Dickinson
	h4-1BB	4B4-1	mIgG1κ	Alexa Fluor 647	BioLegend
	hTim-3	7D3	mIgG1κ	BB515	Becton Dickinson
	hTim-3	7D3	mIgG1κ	BV421	Becton Dickinson
	hTIGIT	A15153G	mIgG2aκ	PE	BioLegend
	hPD-1	EH12.2H7	mIgG1κ	Alexa Fluor 647	BioLegend
	hKLRG-1	2F1/KLRG1	sy ha IgG	FITC	BioLegend
	hNKG2A	REA110	hIgG1	FITC VioBright	Miltenyi
	hNKp46	9E2	mIgG1κ	BV421	BioLegend
pSTAT5 signaling *in vitro*	hCD3	REA623	hIgG1	APC-Vio770	Miltenyi
	hCD8	SK1	mIgG1κ	BV510	BioLegend
	hCD56	REA196	hIgG1	FITC	Miltenyi
	hpSTAT5 (pY694)	47/pSTAT5	mIgG1κ	Alexa Fluor 647	Becton Dickinson
	Isotype control	MOPC-21	mIgG1κ	Alexa Fluor 647	BioLegend

Assay	Reactivity + Target	Clone	Isotype	Fluorophore	Supplier
In vivo PD in tumor-free mice single dose	mCD3	17A2	rat IgG2bκ	BV711	BioLegend
	mCD4	RM4-5	rat IgG2aκ	BV605	BioLegend
	mCD8	53-6.7	rat IgG2aκ	BV421	BioLegend
	mCD45	30-F11	rat IgG2bκ	Alexa Fluor 488	BioLegend
	mCD45R	RA3-6B2	rat IgG2aκ	APC-cyanine7	BioLegend
	mNK1-1	PK136	rat IgG2aκ	PE	BioLegend
	mFoxp3	FJK-16s	rat IgG2aκ	PE-cyanine7	eBioscience
	mKi-67	SolA15	rat IgG2aκ	FITC	eBioscience
In vivo PD in tumor-free mice single vs. multiple dose	mCD3	17A2	rat IgG2bκ	BV711	BioLegend
	mCD4	RM4-5	rat IgG2aκ	BV605	BioLegend
	mCD8	53-6.7	rat IgG2aκ	BV421	BioLegend
	mCD45	30-F11	rat IgG2bκ	APC-cyanine7	BioLegend
	mCD45R	RA3-6B2	rat IgG2aκ	Alexa Fluor 700	BioLegend
	mNK1-1	PK136	rat IgG2aκ	APC	BioLegend
	mKi-67	SolA15	rat IgG2aκ	FITC	eBioscience
	mGranzyme B	GB11	mIgG1κ	PE	Becton Dickinson
In vivo PD in hMUC1-B16.F10 tumor model	mCD3	17A2	rat IgG2bκ	BV711	BioLegend
	mCD4	RM4-5	rat IgG2aκ	BV605	BioLegend
	mCD8	53-6.7	rat IgG2aκ	BV421	BioLegend
	mCD45	30-F11	rat IgG2bκ	APC-cyanine7	BioLegend
	mCD45R	RA3-6B2	rat IgG2aκ	Alexa Fluor 700	BioLegend
	mNK1-1	PK136	rat IgG2aκ	PE	BioLegend
	mKi-67	SolA15	rat IgG2aκ	FITC	eBioscience
	mCD25	PC61	rat IgG1λ	APC	BioLegend
	mFoxp3	FJK-16s	rat IgG2aκ	PE-cyanine7	eBioscience
In vivo PD in humanized DU-145 tumor model	mCD45	30-F11	rat IgG2bκ	PerCP-Cy5.5	BioLegend
	hCD3d	7D6	mIgG2aκ	APC	Invitrogen
	hCD4	RPA-T4	mIgG1κ	BUV395	Becton Dickinson
	hCD8	SK1	mIgG1κ	BV421	BioLegend
	hCD45	HI30	mIgG1κ	FITC	BioLegend
	hCD56	HCD56	mIgG1κ	PE	BioLegend

Supplementary Table S3. TA-MUC1 binding kinetics of GT-00AxIL15

k_{on} [M^{-1} s^{-1}]	k_{off} [s^{-1}]	K_D [nM]
3.02 ± 0.12 E+6	0.96 ± 0.07 E-2	3.18 ± 0.37

Kinetic analysis was performed by switch sense technology (proximity sensing) with immobilized TA-MUC1 peptide and GT-00AxIL15 in solution. Affinity constant KD was determined by the calculation of dissociation (k_{off})/association (k_{on}) rate. Mean ± SD from two experiments.

Supplementary Table S4. Immunophenotyping of PBMCs from tumor cell co-cultures after treatment with GT-00AxIL15 *in vitro*

Marker		CD8⁺ T cells untreated	CD8⁺ T cells GT-00Ax IL15	CD8⁺ T cells fold increase	CD4⁺ T cells untreated	CD4⁺ T cells GT-00Ax IL15	CD4⁺ T cells fold increase
CD25	% positive	1.6	18.6	11.6	5.5	22.8	4.1
OX-40	% positive	2.3	6.5	2.8	13.9	30.4	2.2
ICOS	% positive	21.1	55.7	2.6	50.7	72.4	1.4
4-1BB	% positive	1.2	3.7	3.1	0.5	0.8	1.6
Tim-3	% positive	5	36.2	7.2	2.4	14	5.8
TIGIT	% positive	26.4	33.2	1.3	25.4	27.9	1.1
PD-1	% positive	22.4	36.6	1.6	21.2	33.4	1.6
KLRG-1	% positive	4.8	10.2	2.1	1.6	2.8	1.8
**Marker**		**NK cells untreated**	**NK cells GT-00Ax IL15**	**NK cells fold increase**	**NKT cells untreated**	**NKT cells GT-00Ax IL15**	**NKT cells fold increase**
CD25	%	9.6	79.9	8.3	8.9	72.9	8.2
4-1BB	%	2.6	24.8	9.5	6	11.2	1.9
PD-1	%	2.2	6	2.7	24.9	45.5	1.8
Tim-3	MFI	952	9367	9.8	213	1575	7.4
TIGIT	MFI	1605	4255	2.7	176	251	1.4
KLRG1	MFI	429	563	1.3	182	260	1.4
NKG2A	MFI	804	4715	5.9	141	198	1.4
NKp46	MFI	4532	5348	1.2	173	327	1.9

PBMCs were co-cultured with CaoV-3 tumor cells and 20 nM GT-00AxIL15 for 3 days. Cell surface expression of immune checkpoints was determined by flow cytometry. Results from one representative PBMC donor were shown. MFI = median fluorescence intensity.

Supplementary Table S5. Pharmacodynamic effects of GT-00AxIL15 on peripheral versus tumor immune cell infiltrates *in vivo*

Immune cell population	Organ	Fold increase GT-00AxIL15 (Mean ± SD)
NK cells	Spleen	1.6 ± 0.5
	Tumor	4.6 ± 2.4
$CD8^+$ T cells	Spleen	1.4 ± 0.2
	Tumor	3.7 ± 2.6

Mice with established hMUC1-B16.F10 tumors were treated with a single dose of with 0.5 mg/kg GT-00AxIL15 or vehicle control. Absolute counts of NK and CD8+ T cells were determined by flow cytometry of splenocytes and tumors harvested after 3 days to calculate relative increase by GT-00AxIL15 treatment versus vehicle control (*n*=7-8).
